# A systematic guide for identifying transcription factors that directly regulate the expression of a gene of interest

**DOI:** 10.1101/gr.281154.125

**Published:** 2026-03

**Authors:** Andrew D. Bates, Dawid Grzela, Maciej Studzian, Louise Brennan, Moli Williams, Conor Fawcett, Beth Hammond, Manreen Grewal, Marcin Ratajewski, Lukasz Pulaski, Urszula L. McClurg

**Affiliations:** 1 Institute of Systems, Molecular and Integrative Biology, University of Liverpool, Liverpool L69 7BE, United Kingdom;; 2 Institute of Medical Biology, Polish Academy of Sciences, 93-232 Lodz, Poland;; 3 Bio-Med-Chem Doctoral School of the University of Lodz and Lodz Institutes of the Polish Academy of Sciences, 90-237 Lodz, Poland;; 4 Medical University of Lodz, 90-419 Lodz, Poland;; 5 Department of Oncobiology and Epigenetics, University of Lodz, 90-237 Lodz, Poland

## Abstract

Transcriptional regulation lies at the heart of cellular identity and function, hinging on the precise binding of transcription factors (TFs) and cofactors to gene regulatory elements such as promoters and enhancers. Although it is relatively routine to profile genome-wide DNA binding landscapes of proteins, identifying the specific proteins that bind to, and regulate the transcription of, a particular gene of interest (GOI) remains a persistent experimental and conceptual challenge. This gene-centric question is complicated by the multilayered regulatory environment in which each gene resides, comprising 3D chromatin structure, enhancer–promoter looping, DNA accessibility, histone modifications, and cell state–dependent protein dynamics. In this review, we dissect the strengths, limitations, and biological relevance of current approaches for studying direct protein–DNA interactions, distinguishing between protein-centric and DNA-centric methodologies. We introduce a conceptual matrix of biological relevance, integrating the origin of DNA and protein elements (*cis* and *trans*) to evaluate false-positive and false-negative risks across experimental systems. Moreover, we explore how perturbation strategies—gain and loss of function—can complement steady-state profiling to establish causality in gene regulation. By critically examining both established tools and emerging techniques such as genome editing, synthetic chromosomes, and high-resolution imaging, we provide a practical framework for investigators seeking to uncover direct regulators of specific genes. Our goal is to guide the design of experiments that balance biological relevance, sensitivity, and interpretability to ultimately answer a deceptively simple question: What TFs directly regulate the expression of my GOI?

Cellular processes are regulated by transcription machinery binding the promoters and enhancers of target genes to activate gene expression. This is followed by a cascade of RNA processing and protein translation, allowing for the newly expressed protein to execute its function, which in turn may contribute to cell signaling. Consequently, one of the main questions that often arises in research is what transcription factors (TFs) regulate the expression of my gene of interest (GOI) directly on its promoter. However, answering this is not straightforward. Genes exist within multilayered microenvironments of proximal and distal promoter–enhancer interactions, DNA accessibility, histone post-translational modifications (PTMs), and DNA looping and high-order chromatin structure (Panigrahi and O'Malley 2021; Karr et al. 2022; Chen et al. 2024b). Furthermore, the DNA interactome at a particular gene locus is controlled by the availability of the transcription machinery, its correct localization, transcriptional complex formation, and the PTM profile of regulatory proteins. To investigate direct DNA–protein interactions, scientists can either isolate a protein of interest and identify all of the DNA sequences capable of binding to it or, conversely, use a DNA sequence of interest to identify proteins that are capable of binding to it. These two approaches answer fundamentally different questions: The first one allows us to discover the DNA binding profile of a protein of interest; the second one is aimed at identifying proteins that may directly regulate a GOI.

Protein-centric approaches were initiated by the development of chromatin immunoprecipitation (ChIP) in 1984 followed by ChIP-qPCR and ChIP-seq, which allowed scientists to probe the full palette of DNA molecules bound by proteins of interest. A ChIP experiment begins by using formaldehyde to chemically cross-link DNA and protein molecules (Das et al. 2004). Nuclei are isolated, and the chromatin (which now contains fixed protein–DNA complexes) is sonicated, or another method of shearing it into fixed-average-length DNA fragments is applied. DNA fragments bound to proteins are incubated with antibodies specific to a protein of interest and antibody–protein complexes are precipitated using beads. To focus on individual candidate genes predicted to be bound by the protein of interest, precipitated DNA can be analyzed using qPCR with primers designed specifically against the regulatory sequences of the GOIs. Alternatively, in ChIP-seq following precipitation, cross-links are reversed, and the released DNA can be sequenced to identify all DNA sequences bound to the protein of interest using high-throughput platforms (Park 2009). Standard controls include comparing this immunoprecipitation to DNA binding by nonimmunized IgG raised in the same species as the protein targeting antibody, using qPCR primers designed against nonregulatory regions of the gene when enrichment is not expected, and ensuring DNA shearing with fragments that are not too long in order to prevent nonspecific GOI identification. ChIP has had a revolutionary impact on our understanding of biology and has spearheaded clinical translation. Complete genome-wide ChIP-seq for a TF can be mapped within the cells and tissues in an efficient and timely manner with novel technologies.

However, complications arise when we pose the DNA-centric question: which proteins are bound to, and directly regulate, a GOI. Initial approaches consisted of electrophoretic mobility shift assays (EMSA) (Hellman and Fried 2007). In an EMSA, labeled probes corresponding to the DNA of interest are synthesized or isolated and are consequently incubated with a purified protein of interest or with a mixture of proteins (e.g., a nuclear extract). To determine if direct binding of DNA and protein has occurred, reaction is separated on a nondenaturing agarose or polyacrylamide gel to study if there has been a shift in DNA mobility caused by increased size owing to protein binding. To confirm the identity of DNA-bound protein, mobility supershift by specific antibodies may be tested. However, EMSA lacks the majority of biological context. Many proteins are capable of binding naked DNA; however, within the cellular environment they might not be localized to the nucleus, preventing this binding. Furthermore, in the cell the DNA sequence of interest might not be accessible owing to chromatin compaction or PTMs as well as competition from other binding proteins. Consequently, EMSA can be applied to rule out DNA–protein interactions: If a protein is not able to bind the sequence of interest in an EMSA assay, it is unlikely that this interaction occurs in nature; however, positive binding in an EMSA requires further confirmation of interaction within the cellular environment.

When selecting a method to study DNA–protein interactions, it is crucial to consider various experimental parameters. In molecular genetics, DNA is referred to as the *cis*-element, whereas the protein that binds to it is the *trans*-element. The biological relevance of these elements must be evaluated individually. Analyzed DNA can be a synthetic, naked DNA sequence of in vitro origin; an artificial chromosome introduced into a cell (exogenous DNA); or endogenous DNA within the cell's genome. Exogenous DNA loses characteristics such as genome compaction and accessibility, whereas endogenous DNA allows for in cellulo analysis under physiological conditions. Similarly, proteins can be purified and studied in vitro, expressed exogenously from a plasmid, or endogenously produced within the cell from genomic DNA. Although plasmid-based expression allows for controlled experiments, it lacks physiological regulation of protein levels. Using endogenous protein ensures a physiological setting for studying DNA–protein interactions. These variations create a biological relevance matrix, in which *cis*-elements and *trans*-elements can range from fully artificial to completely physiological (Fig. 1). This choice of system affects the likelihood of false positives and false negatives. In vitro experiments carry a high risk of false positives, as binding may occur under artificial conditions but might not be reproduced in a cellular environment owing to factors like protein compartmentalization or DNA inaccessibility. However, false negatives are less likely. Endogenous cellular experiments have a lower risk of false positives owing to the physiologically relevant context. However, they are more prone to false negatives, as protein–DNA interactions may vary depending on the cell type, cell cycle stage, or environmental stressors and can be missed depending on the experimental setup. It is impossible to account for all these variables in a single experiment.

**Figure 1.**
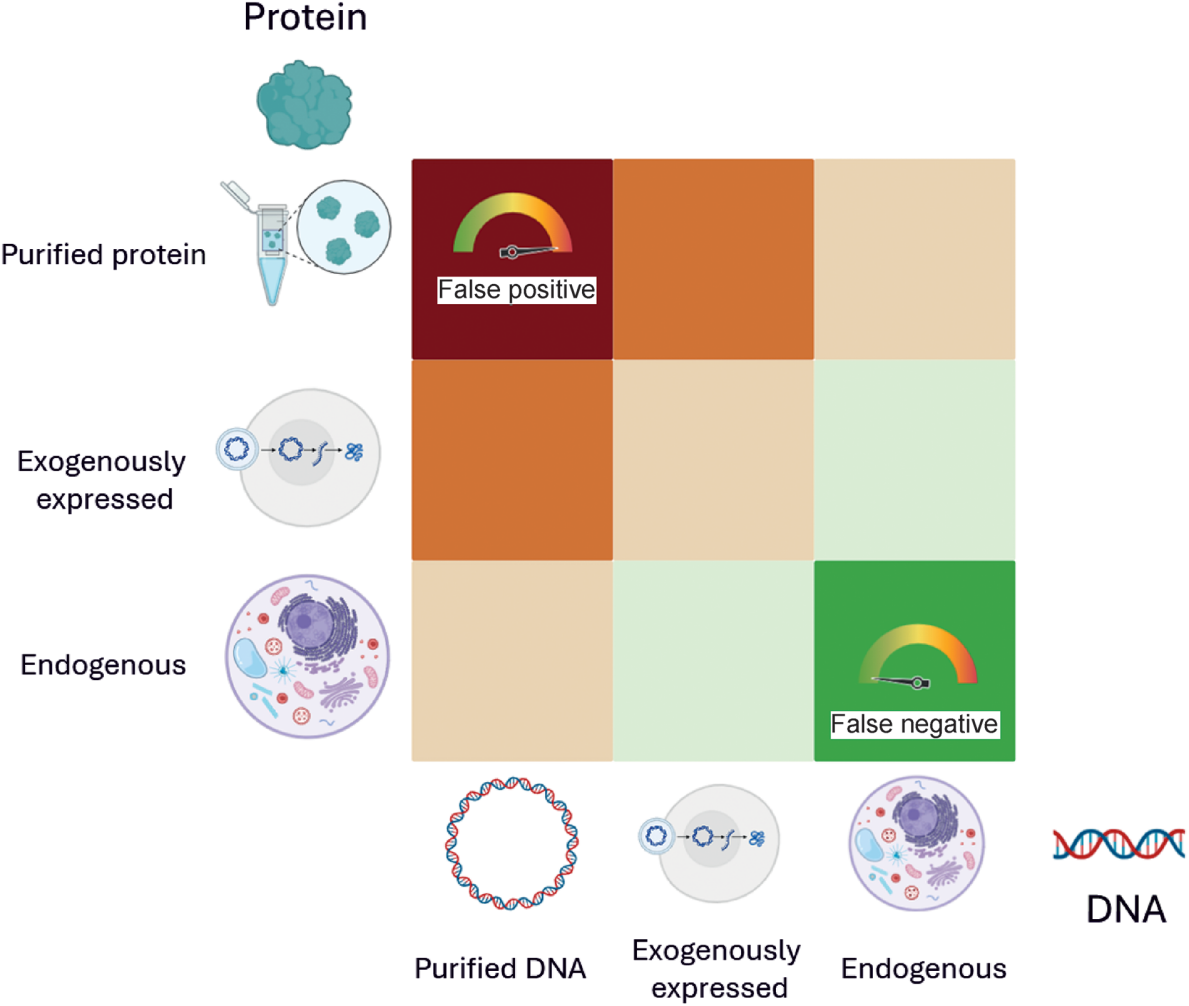
Matrix of biological relevance in DNA–protein interaction studies. Experimental approaches to study DNA–protein interactions vary in their use of *cis*-elements (DNA) and *trans*-elements (proteins). The matrix illustrates combinations of DNA substrates (in vitro naked DNA, exogenously introduced DNA, and endogenous chromatin) and protein sources (purified, plasmid-expressed, or endogenously produced) arranged by increasing physiological relevance along the diagonal from *bottom right* to *top left*. Cells are color-coded to reflect biological relevance, ranging from low (red) to high (green). Systems using purified proteins and naked DNA are prone to false positives, whereas fully endogenous systems, although biologically informative, carry a higher risk of false negatives owing to contextual dependencies such as chromatin state, cell cycle stage, and transcriptional noise. It is critical to carefully select experimental configurations to balance control, sensitivity, and physiological accuracy when investigating transcription factor–DNA interactions, although of course no method is free of risk; for example, EMSA can also generate false negatives (e.g., false negative for binding when a cofactor would be required in the cell environment).

Beyond biological relevance, it is also important to distinguish between methods that study the steady state of the cell versus those that introduce experimental perturbations for comparative analysis. Some techniques rely on genetic modifications to reveal functional differences. Gain-of-function approaches introduce new elements, such as exogenous proteins, activating mutations, plasmids, artificial chromosomes, or knock-in modifications. Conversely, loss-of-function approaches remove specific factors using mutations, siRNA silencing, or gene-editing techniques. These modifications can be applied to either the DNA or the protein, further influencing experimental outcomes.

Although well-established approaches have been developed to determine DNA binding patterns for a protein of interest, because of a lack of an established, and universally accepted, methodology for identifying direct regulators of a GOI, there is a risk of wasting time and money by taking false or indirect routes that will not answer the question at issue. In this review, we discuss advances that have been made to address this methodological challenge, as well as technical and biological limitations of existing technologies. We compare existing methods, provide a guide for considerations that need to be made during study design, and highlight novel approaches that are becoming possible with recent developments in genome editing as well as high-resolution imaging.

To answer which proteins are bound to and directly regulate the transcription of a GOI, we need to know the following:

– What are the regulatory elements, especially promoters and enhancers, of my GOI?
– What proteins bind to the regulatory elements of my GOI?
– Which of the proteins bound to my GOI directly regulate its expression?

## Computational prediction of regulatory sites and TF binding sites

A eukaryotic gene typically consists of regulatory elements such as distal enhancers, which can act over long distances; a proximal promoter containing key TF binding sites; a core promoter surrounding the transcription start site (TSS) where the preinitiation complex assembles, followed by the coding region (exons and introns); and ending with a terminator and polyadenylation signals that ensure proper transcript processing and stability. Before we can determine proteins that directly regulate GOI expression, we must identify the regulatory DNA sequences relevant to the GOI such as its promoter(s) and enhancer(s) (Zhang et al. 2022). Although many tools exist for this purpose, newcomers should understand the benefits and limitations of in silico approaches. With advanced, often algorithmically opaque, machine learning tools now available, a cautious approach can save time and effort. It is critical to appreciate both the biochemistry of TF-DNA binding specificity and the probabilistic nature of tools that predict it. TF binding depends on a number of non-sequence-dependent factors in addition to the local DNA sequence information that prediction tools are based on, leading to an inordinately high level of false-positive predictions that the user must expect and take into account. Sequence-independent factors include DNA accessibility, competition from other binders, and TF localization regulation. This knowledge helps biologists generate testable hypotheses without overrelying on potentially misleading predictions.

### Prediction of potential regulatory sites

This review focuses on gene-specific transcriptional regulation via direct TF binding to regulatory DNA sites. The initial task is thus to locate candidate DNA regions potentially involved in TF binding, and this starts with identifying the TSS, the RNA polymerase recruitment point. TF binding sites near the TSS form the promoter, whereas more distant ones are typically called enhancers (Bateman and Johnson 2022). Some genes have multiple functional TSSs, which complicates regulatory analysis, so alternative promoters must be considered (Nepal and Andersen 2023). For well-studied organisms like humans, genome-wide data are accessible and can assist with TSS identification. For lesser-known genomes, predictive tools use core promoter sites to infer probable TSS locations (Adato et al. 2024). These predictions should be verified by experimental data, with attention to possible alternative TSSs in introns or distant upstream regions (Alfonso-Gonzalez and Hilgers 2024). Widely-used tools for TSS prediction include the trainable *ab initio* software TSSFinder (de Medeiros Oliveira et al. 2021) tailored for broad, nonselective application in any eukaryotic organism, TSSPlant (Shahmuradov et al. 2017) designed for plant TSS prediction, and machine learning-enhanced options like DeePromoter (Oubounyt et al. 2019) designed exclusively for mammalian TSSs. Newer CamoTSS specifically calls TSS from actual RNA-seq data (Hou et al. 2023).

Most regulatory events occur at TF binding sites some distance from the TSS, and predicting these is crucial in computational genetics. TF binding operates within a topologically associating domain typically upstream of the TSS but sometimes within the gene. However, predicting the boundaries of this domain in silico is challenging; researchers often analyze an arbitrary sequence length surrounding the TSS or rely on database-mined experimental data. Sequence conservation analysis can help identify important regulatory sequences (Stark et al. 2007).

At least 40%–50% of human genes are subject to some degree of regulation by DNA methylation, depending on factors such as cell type, developmental stage, and environmental conditions (Gardiner-Garden and Frommer 1987; Jaenisch and Bird 2003; Illingworth and Bird 2009; Loyfer et al. 2023). CG-rich sequences are predominantly found in the 5′-flanking regions of genes and are bound by TFs such as Sp/Kruppel-like factor (Kaczynski et al. 2003; Suske et al. 2005). Methylation of these sequences prevents the binding of TFs, leading to the recruitment of proteins associated with methyl-CpG binding (Du et al. 2015), which in turn inhibits transcription. Several tools are available for predicting the presence of CpG islands in DNA sequences. Examples include the CpGplot tool from the European Molecular Biology Open Software Suite (EMBOSS) (Rice et al. 2000), the CpG Island Searcher (Takai and Jones 2003), CpGProD (Ponger and Mouchiroud 2002), and CpGPAP (Chuang et al. 2012).

Predicted regulatory sites can be validated experimentally using reporter gene assays. In this assay, the transcriptional activity of a predicted promoter, or an enhancer, of a specific GOI can be investigated using plasmids that include the predicted regulatory sequence upstream of a reporter gene (for promoter testing) or upstream of a known promoter linked to a reporter gene (for enhancer testing), encoding an easily assayed protein such as GFP, LacZ, or luciferase. Reporter assays involve DNA transcription within cells to evaluate their regulatory activity (Romanov et al. 2021), if the regulatory sequence is functional, the reporter gene is expressed/induced and the cellular concentration of the gene product is measured (Smale 2010).

### TF binding prediction

TFs bind DNA in a sequence-specific manner based on motifs, with each binding site having a consensus sequence that indicates prerequisites of functional binding. Binding affinity is usually degenerate, with multiple sequence variations binding equally well (Stormo and Zhao 2010). Many TFs, especially in eukaryotes with larger genomes, have low specificity, leading to high false positives in binding site predictions. Short, degenerate DNA sequences matching a TF motif often occur by chance; consequently, most motif matches in the genome are not functional binding sites (Wasserman and Sandelin 2004). This highlights that a sequence matching a position weight matrix (PWM) does not necessarily indicate actual TF binding in vivo. Functional binding depends on additional context, including chromatin accessibility, the presence of cofactors, 3D genome architecture, cell type, and other regulatory factors—not just the DNA sequence itself. Consequently, predictive tools must tackle the challenge of filtering out nonfunctional sites, a task still evolving, with new algorithms frequently published after four decades of research (Stormo et al. 1982).

The classical method for dealing with motif degeneracy is to compare the regulatory sequence in a GOI to a PWM, which scores TF binding potential at each site based on experimentally derived probabilistic data (Mathelier and Wasserman 2013). PWMs, often displayed as sequence logos, which are bar graphs with bars made of nucleotide symbol letters, help illustrate binding motif conservation. Energy matrices, an alternative to PWMs, account for binding energy contributions. Still, PWMs remain dominant owing to early statistical approaches preceding the availability of structural data. PWM-based predictions depend on databases like TRANSFAC (Matys et al. 2006) and JASPAR (Khan et al. 2018). Modern high-throughput techniques that experimentally identify protein–DNA binding, such as ChIP-seq, SELEX, and protein-binding microarrays, have significantly improved database quality (Jolma et al. 2013). However, the human genome has nearly 2000 transcription regulators, many of which share consensus sites, adding complexity to predictions (Lambert et al. 2018).

Despite improvements, PWM-based predictions suffer from high false positives owing to oversimplified assumptions, such as treating nucleotides as independent. Dinucleotide matrices and *k*-mer approaches address some limitations (Siddharthan 2010), but factors like overlapping TF binding sites, cooperative binding, and DNA shape further complicate predictions (Srivastava and Mahony 2020). The chromatin context also affects TF binding, as many consensus sites may be inaccessible owing to histone-bound heterochromatin (Zhu et al. 2018). Chromatin openness strongly correlates with TF binding potential (Thurman et al. 2012), and DNA methylation and local DNA shape also influence binding (Yin et al. 2017). Lastly, it is important to remember that TFs can regulate genes without directly binding DNA, through complexes with other TFs (tethering) (Yamada et al. 2019). This effect must be experimentally verified to fully understand GOI regulation.

### A summary of TF binding prediction for nonadvanced users

Starting with established PWM-based tools like MatInspector (Cartharius et al. 2005) can be effective. More advanced options, such as PSCAN (Zambelli et al. 2009) and MEME (Bailey et al. 2015), improve reliability by analyzing binding site enrichment. Tools like REUNION (Yang and Pe'er 2024), Pando (Fleck et al. 2023), and TRIPOD incorporate chromatin data, enhancing specificity. TFBShape and CRPTS account for DNA shape influences (Yang et al. 2014). Given the inherent challenges, modern prediction tools often incorporate machine learning to enhance traditional methods. Early tools like hidden Markov models and support vector machines have given way to neural networks, such as convolutional and recurrent models, which power tools like DeepBIND (Alipanahi et al. 2015) and DeepTF (Bao et al. 2019). Although user-friendly classical tools remain popular, newer models like DeepReg (Ledesma-Dominguez et al. 2024) and DeepGRN (Chen et al. 2021a) offer improved specificity by integrating chromatin accessibility.

Recent deep learning tools offer incremental improvements and may appeal to advanced users. However, for practical GOI analysis, especially in laboratories with limited IT resources, classical algorithms are often sufficient. Combining tools with awareness of their limitations allows researchers to design experiments with high-confidence hypotheses. However, one of the major steps when trying to identify functional regions of a gene involves systematic perturbation of its regulatory DNA through deletion, linker-scanning, or site-directed mutagenesis. These approaches enable precise mapping of essential *cis*-elements, such as promoters, enhancers, and TF binding sites, by directly testing the impact of sequence alterations on transcriptional activity.

## Data mining genome-wide studies

Although computational prediction alone cannot explain the transcriptional regulation of your GOI, this does not mean that you have to do all of the “wet-laboratory” experiments yourself: Data mining is a crucial step in developing your hypotheses. Proliferation of easily accessible, large-scale, high-throughput studies provides fertile ground for analyses. The sources of useful data for inferring TFs that might regulate a GOI include

– Proximity to experimentally proven TSSs.
– Chromatin landscape indicating open chromatin or, more specifically, epigenetic features known to be linked with enhancers.
– TF binding to DNA sequences within the GOI locus, preferably within suspected regulatory regions.
– Positive correlation between expression of regulators (TFs) and the GOI product at the protein level; this can be determined by comparing different tissues/cell types or by observing fluctuations at the single-cell level.
– Instances when disruption, or perturbation, of the regulator (TF) causes disruption/perturbation of the GOI product: This may mean correlating the GOI expression with downregulation or disruption of TF, experimental disruption of TF binding site in the regulatory element, or phenotype (e.g., disease)-linked noncoding variants in regulatory sequences.
– Conservation of regulatory mechanisms in GOI orthologs in other organisms.

### Identifying regulatory regions

To determine TSS(s) by data mining, whole-genome 5′-RACE, CAGE, or RNA-seq experiments are readily available either through genome browsers or through dedicated databases like FANTOM5 (Kawaji et al. 2014) or DBTSS (Suzuki et al. 2015). However, for genes expressed at a low level, whole-genome experiments might not have enough coverage to reliably pinpoint TSS(s).

Several databases provide comprehensive DNA methylation data derived from high-throughput sequencing, including single-base resolution methylation levels for individual CpG sites across the genome. These resources also annotate hypomethylated regions commonly linked to gene promoters, as well as allele-specific methylation patterns relevant to genomic imprinting. Notable examples include MethBase (Song et al. 2013), MethBank (Zhang et al. 2023), EWAS Atlas (Li et al. 2019b), Human Epigenome Atlas (Milosavljevic 2010), and iMethyl (Hachiya et al. 2017).

The euchromatic signatures present in enhancer-rich stretches of chromosomes include increased DNA accessibility to nucleases and modifying enzymes (e.g., measured by DNase I hypersensitivity), high nucleosomal turnover (e.g., high levels of rapid-turnover histones H2A.Z and H3.3), specific epigenetic markers (e.g., high ratio of H3K4me1 and H3K4me2 to H3K4me3, high ratio of 5hmC to 5mC, enrichment of H3K27ac), chromatin loops in direct contact with the core promoter (enrichment of cohesin complexes, peaks in chromatin proximity assays such as Hi-C or ChIA-PET), and the appearance of randomly, bidirectionally transcribed nonpolyadenylated cognate eRNA (Long et al. 2016). Most accessible repositories for the entry-level molecular geneticist with a limited number of GOIs are those that collate and integrate many different sources of data and databases because ease of use and reliability are key.

Three types of tools should be applied in parallel:

– Genome browsers with multiple annotation tracks, especially the unrivalled University of California at Santa Cruz (UCSC) browser (Perez et al. 2025)
– Nonspecialized hyperlinked databases of genetic and genomic information, such as the broad-scope Harmonizome (Rouillard et al. 2016), which is intuitive in use, or GeneCards (Stelzer et al. 2016), which is more limited with regards to gene regulation but abundant in information about gene function and links to practical research tools
– Tools oriented specifically toward gene regulation, which allow reliable data mining, such as the excellent Gene Transcription Regulation Database (Kolmykov et al. 2021), the main advantage of which is seamless integration of ChIP-seq, ChIP-exo, DNase-seq, and MNase-seq chromatin status with ChIP-derived data regarding specific TF binding.

Most large-scale data come from two major, multidecade projects, ENCODE (Luo et al. 2020) and FANTOM (Abugessaisa et al. 2021), both of which make their data available directly, but accessing it is much easier via the above-mentioned browsers and databases. If data for your GOI turn out to be limited or if there is reason to suspect cell-specific epigenetic factors at play, more focused chromatin accessibility databases should be studied, such as deepBlue (Albrecht et al. 2016), which is unfortunately available only via GitHub, or i-*cis* Target, which has a useful option of selecting the types of experiments to browse (e.g., ChIP-seq, FAIRE-seq, or ATAC-seq) (Verfaillie et al. 2015). Additionally, ATACdb, is specific to ATAC-seq data (Wang et al. 2021), whereas the 3D Genome Browser (Wang et al. 2018) compiles chromosome conformation capture (3C), HiC, and ChIA-PET data on chromatin loops.

### Direct TF binding

There are two main original sources of data on specific TF binding to the regulatory sequences of your GOI: major genome-wide programs (such as the above-mentioned ENCODE or FANTOM, which are especially rich in data on ncRNAs) and individual studies that concentrate either on a limited number of TFs or on specific groups of genes. Once again, the best practical path to easily accessing these types of data is via curated integrating databases, especially Harmonizome and GTRD. It is important to remember that although these are experimental data that detected actual TF binding to a specific locus or site, this does not necessarily mean that the TF has an important function in GOI regulation, especially not in your favorite cell type/condition/treatment. However, this is probably the strongest hypothesis-generating tool available. More specialized tools for this approach include the Peak Browser at ChIP Atlas (Oki et al. 2018) or browsing ChIPBase (Huang et al. 2023), which is especially strong for ncRNAs but can also be used for mRNA genes. The Eukaryotic Promoter Database ExPASy has the useful Mass Genome Annotation Archive, which includes ChIP-seq TF annotation (Dréos et al. 2018).

### Secondary sources that suggest regulators of your GOI

Correlating expression of your GOI at the RNA level with known TFs or other target genes, for which regulatory pathways have previously been elucidated, is an important way to generate testable hypotheses. Cscan is a simple tool for finding common regulators of several GOIs (Zambelli et al. 2012). COXPRESdb (Obayashi et al. 2019) and the somewhat more cumbersome SEEK (Zhu et al. 2015) were created to study coexpression relationships across a broad spectrum of data, including various model organisms, making it possible to search for a similar function. The richest source of potentially unexpected perturbation-based data comes from correlating genetic variability in candidate regulatory regions with known phenotypes. The GTEx Consortium et al. (2020) has analyzed variations in potential regulatory sequences and their association with expression quantitative trait loci (eQTLs) to form the most comprehensive resource of this type, accessible through the UCSC Genome Browser. If your GOI and/or candidate TF are known to be expressed in human cancer, two useful tools that are relevant are DepMap, which allows regulator/target coexpression analysis (Tsherniak et al. 2017), and the Xena Browser (Goldman et al. 2015), which can also be used to correlate variation with expression (both of GOI and of candidate TF). It is, however, important to remember that DepMap does not validate successful knockout and is susceptible to splice variant “blindness.” Human cancer is especially rich in variations, including coding region mutations of TF genes; these features can sometimes offer similar insights as knockdown/knockout experiments. The Eukaryotic Promoter Database has the SNP2TFBS subdatabase linking genetic variants to known, or potential, TF binding sites (Kumar et al. 2017).

One should not discard more remote sources to infer potential TFs that could regulate your GOI: Orthologous genes in better-studied organisms are often a goldmine of information for designing initial experiments. Analyze databases of regulatory regions in model organisms that combine data and predictions: The best-known (and available via many integrator tools) is ReMap (Hammal et al. 2022), but an interesting alternative is CistromeDB (Zheng et al. 2019). There are also time-honored genomic resources for specific model organisms, such as the *Saccharomyces* Genome Database (Cherry 2015), *Arabidopsis* Gene Regulatory Information Server (Yilmaz et al. 2011), WormBase (Sternberg et al. 2024), and FlyBase (Jenkins et al. 2022). Finally, sometimes little known publications contain gems of prepublished experimental data that should not be overlooked; carefully curated databases compile as many of them as possible, including JASPAR (Rauluseviciute et al. 2024) and ORegAnno (Lesurf et al. 2016), both available via the USCS Genome Browser. MSigDB contains curated gene sets from the literature; the most useful ones for the purpose described here are the regulatory target gene set and the chemical and genetic perturbation gene set (Liberzon et al. 2015). Among modern AI-based tools, TRRUST finds co-occurrences of mentions of terms (e.g., TFs and GOIs) in published literature (Han et al. 2015).

### Practical gene regulation data mining for nonadvanced user

When you first approach the question of what regulates your GOI transcriptionally, you should start by compiling available information on chromatin landscape (especially regulatory region hallmarks) and TF binding from genome browsers (e.g., UCSC Genome Browser) and user-friendly databases (e.g., Harmonizome). For most genes and organisms, this should provide plenty of starting points for planning confirmatory experiments. If the available data appear insufficient, contradictory, or unsuitable, it may be necessary to delve into specialized databases or explore additional sources, such as perturbation studies, coexpression patterns, or homology analyses with other organisms. It is crucial to keep in mind that all in silico approaches, both predictive and based on data mining, are never sufficient to determine GOI regulators owing to the complexity of transcriptional regulation and intricacies of protein–DNA binding. Their main use is for generating experimentally testable hypotheses for further research.

## Experimentally validating TFs by measuring transcription from the GOI

When dissecting transcriptional regulation, the first experimental point of interest is the process of transcription itself and its immediate product, the RNA transcript. The ability to detect whether the GOI is being transcribed in a cell or in a group of cells provides a direct readout that can be tested by introducing experimental perturbations to TF level or activity. Directed modifications to genomes in experimental models are becoming essential for elucidating how specific genes, their regulatory elements, and other genome-related components influence physiological processes. Gene editing encompasses various methodologies and involves the targeted insertion, deletion, modification, or replacement of DNA sequences at precise locations within the genome. In the simplest terms, if we can accurately detect transcription of a GOI by comparing transcript levels between wild-type (WT) cells and cells depleted of a predicted TF for the GOI, we can validate if the TF in question regulates the GOI. Similarly, we can compare transcription of the GOI between different cellular conditions such as hypoxia, starvation, DNA damage, etc., to determine the cellular context required for GOI expression.

Originally the nuclear run-on technique was developed to detect the emergent transcripts that are in the process of being produced from the GOI. In this approach, intact nuclei are flash-isolated from cells, depriving them of ribonucleotide substrates for transcription. This stalls RNA polymerases on genes that were in the process of being transcribed. Subsequently, transcription is restarted and allowed to run to completion by adding exogenous-labeled ribonucleotides that are incorporated into completed transcripts and allow for their identification, for example, by hybridization-based techniques with radiolabeling (Smale 2009). This approach was recently improved by including modified nucleotides that allow for enrichment of the emergent transcripts by affinity isolation. Some examples of modified nucleotides include bromouridine, which can be immunoprecipitated by specific antibodies (global run-on assay) (Core and Lis 2008), or biotinylated cytosine, which can be bound to streptavidin-based binders (precision run-on assay) (Mahat et al. 2016). The resulting transcripts are usually subjected to RNA-seq, but transcription from an individual GOI can also be quantified by qPCR. This method is compatible with experimental manipulations which can identify cellular conditions and/or TFs that are capable of switching GOI transcription on or off.

The challenge of run-on analysis lies in the need to isolate nuclei from their cellular environment. Alternative methods, which allow the relative quantification of nascent transcripts (freshly produced RNA) in cellulo, have emerged. Initial approaches concentrated on stopping transcription with pharmacological inhibitors of RNA polymerase (such as actinomycin D). Currently, the mainstream approaches include the use of click chemistry to isolate transcripts with incorporated modified nucleotides provided as a pulse to cells (Palozola et al. 2017) as well as those that use fluorescent in situ hybridization (FISH) and advanced imaging to visualize emergent transcripts at the single-cell (or sometimes single-GOI) level (Salataj et al. 2023). Recently, a sophisticated method of quasi-immediate visualization of transcribed genes has been developed in the form of nascent RNA tagging. This method requires the GOI modification (endogenous or exogenously introduced, e.g., on an artificial chromosome) by insertion of an aptamer-like sequence (e.g., MS2 or PP7), which upon transcription folds into a tertiary structure that binds a fluorogenic dye with high affinity (Chubb et al. 2006). This powerful technique can be combined with superresolution and multicolor imaging to provide single-allele resolution of GOI expression (Hocine et al. 2013). However, as mentioned above, modification of cell state is required for this type of observational approach to provide answers regarding potential GOI regulators.

Alternatively, changes in TF activity and transcriptional regulation can be inferred from experimentally measured changes in chromatin accessibility at carefully selected gene loci. For this purpose, Förster resonance energy transfer combined with fluorescence in situ hybridization (FRET-FISH) was recently developed (Mota et al. 2022). This method takes advantage of the sensitivity of FISH and the spatial resolution of FRET. FRET-FISH uses DNA probes labeled with donor and acceptor fluorophores that are designed to bind to specific DNA sequences. In close proximity, energy transfer occurs from the donor to the acceptor, which can be quantified to assess the distance between the two probes. This distance is inversely related to chromatin compaction; a high FRET efficiency indicates close proximity (and thus compacted chromatin), whereas low FRET efficiency indicates a more relaxed chromatin state. FRET-FISH can measure chromatin compaction at selected gene loci, providing insights into the accessibility of a GOI. Chromatin compaction measured by FRET-FISH was shown to correlate strongly with chromatin accessibility data obtained from ATAC-seq (Mota et al. 2022). FRET-FISH can also detect changes in chromatin compaction in response to drug treatments, during different phases of the cell cycle, and as cells age. Beyond chromatin compaction, FRET-FISH could in the future be adapted to study enhancer–promoter interactions and chromatin loop organization with greater resolution compared with straightforward FISH or aptamer-tagging techniques used so far (Nazarova and Sexton 2026), making it a versatile tool in single-cell genomics.

Traditionally, steady-state mRNA levels have been assessed using qPCR or RNA-seq, but these approaches capture both primary and secondary responses and are influenced by RNA stability. To address this limitation, a variety of nascent RNA profiling techniques have been developed that monitor transcription as it occurs. Pioneering work introduced methods such as global run-on sequencing (GRO-seq) (Core and Lis 2008) and precision nuclear run-on sequencing (PRO-seq) (Kwak et al. 2013), which measure the location and activity of engaged RNA polymerase II (Pol II) genome-wide. These approaches provide a high-resolution view of transcription initiation, pausing, and elongation, enabling rapid detection of transcriptional changes following TF perturbation.

Other methods survey Pol II–associated chromatin-bound RNAs, such as chromatin-associated RNA-seq (ChrRNA-seq) (Bell et al. 2018) or transient transcriptome sequencing (TT-seq) (Schwalb et al. 2016), which enrich for nascent transcripts and thereby sharpen the link between TF activity and transcriptional outcome. Together, these nascent RNA methodologies offer a more direct readout of transcriptional regulation, making them particularly powerful for validating whether a TF truly governs expression of a given gene.

## Experimentally validating TFs by measuring GOI transcript levels

Because the first outcome of a TF activating expression from the GOI is transcription, which leads to production of cognate RNA, measuring steady-state RNA levels is an obvious proxy for gene expression. One of the most direct approaches to assess whether a candidate TF regulates a GOI is to perturb TF abundance and measure downstream transcriptional consequences. Several complementary technologies exist for TF depletion, each with distinct strengths and caveats.

RNA interference (RNAi), typically in the form of short interfering RNAs (siRNAs) or short hairpin RNAs (shRNAs), has been widely used to reduce TF levels by promoting degradation of the corresponding mRNA. Although inexpensive and relatively easy to implement, RNAi often results in incomplete knockdown and can suffer from off-target effects, which complicate interpretation of transcriptional changes.

Genome editing approaches, such as CRISPR–Cas9-mediated knockout (Nakamura et al. 2021), enable permanent removal of a TF. Knockouts are powerful for defining essential regulatory roles but can be confounded by compensatory adaptation during cell culture or development, particularly when TFs are essential for cell survival or identity. CRISPR interference (CRISPRi), which uses a catalytically inactive Cas9 fused to a repressor domain, allows reversible transcriptional silencing of the TF gene without DNA cleavage, providing a tunable alternative.

Conditional protein depletion systems have become increasingly popular for dissecting TF function with higher temporal resolution. Degron-based strategies, such as the auxin-inducible degron (AID) (Nishimura et al. 2009), the dTAG system (Nabet et al. 2018), or Halo- (Tovell et al. 2019) and SMASh-tags (Chung et al. 2015), allow acute and reversible depletion of TF proteins in response to a small molecule. These methods are particularly valuable because they bypass transcriptional adaptation and permit rapid TF loss, thereby minimizing indirect or secondary effects. Compared with RNAi or CRISPR knockout, degrons can thus reveal more immediate transcriptional dependencies on the TF of interest.

In practice, combining depletion strategies with the precise transcriptional readouts discussed in this section provides a robust framework for validating direct TF–GOI relationships. Acute depletion approaches, especially degron systems, are now considered state-of-the-art for verifying TF function because they best preserve the causal connection between factor presence and gene regulation.

Northern blotting (NB) is a well-established technique that enables the detection and quantification of RNA transcripts of interest. This method involves separating RNA molecules by size using gel electrophoresis, followed by immobilization onto a membrane for hybridization with a labeled sequence-specific probe (He and Green 2013). Despite its reliability, NB has notable limitations, including low sensitivity, labor-intensive protocol, and the requirement for relatively large amounts of RNA. Importantly, NB does not provide insights into the spatial localization of transcripts or post-transcriptional modifications.

RNase protection assay (RPA) offers a more sensitive approach with the concomitant ability to determine TSSs by using sequence-specific DNA probes together with single-strand-specific nucleases to preserve hybridized RNA fragments while degrading unprotected single-stranded RNAs (Zhao et al. 2020). This method enables precise quantification of RNA abundance and mapping initiation sites, crucial for identifying promoter regions and TF binding sites. However, RPA is technically demanding, requiring careful probe design and optimization to minimize background noise. Additionally, it is less suitable for high-throughput analysis.

Historically microarrays were widely used to quantify transcript levels across the genome by hybridizing fluorescently labeled cDNA to thousands of predefined DNA probes immobilized on a solid surface. Each probe was designed to be complementary to a specific transcript, allowing its abundance in a sample to be inferred from the intensity of the fluorescent signal. However, the reliability of the microarray data was highly dependent on probe design; poorly designed probes could lead to cross-hybridization, in which nontarget transcripts bound to probes with partial sequence similarity, thereby generating false-positive or ambiguous signals.

Because probe sequences had to be predetermined, microarrays could only detect transcripts for which corresponding probes were included on the array. This meant that novel or poorly annotated genes were often completely missed, especially in early versions of microarrays based on incomplete genome annotations. Moreover, differential splicing, overlapping transcripts, or polymorphisms could further complicate interpretation, as probes were not always isoform specific.

Expression profiles generated by microarrays were thus limited to what the array was “designed to see,” making it a hypothesis-limited rather than truly discovery-based method. Additionally, probe performance could vary significantly between transcripts, necessitating careful normalization and often validation by qRT-PCR or NB. Despite these limitations, microarrays enabled high-throughput transcriptomic comparisons across conditions and were instrumental in defining early gene expression signatures in development and disease, whereas they have been largely retired following high-throughput RNA-seq development. However, mining existing databases of microarray studies following candidate perturbation can generate valid hypothesis regarding GOI regulators, but it is critical to keep in mind the limitations of this technique.

RT-qPCR can be employed to validate in silico predictions and determine whether the suspected TF plays a role in the transcription of the GOI. Total mRNA is first reverse-transcribed into complementary DNA (cDNA); this cDNA template is used for the quantitative PCR or real-time PCR reaction (qRT-PCR). Specific target fluorescent DNA-binding probes or fluorescent dsDNA-binding dyes (nonspecific used with target-specific primers) are incorporated during the PCR reaction, so that the increase in emitted fluorescence proportionally correlates with the GOI transcript quantity (Nolan et al. 2006; Green and Sambrook 2018).

Digital droplet PCR (ddPCR) is a highly sensitive method used to precisely quantify transcripts by partitioning a PCR reaction into thousands of nanoliter-sized droplets, each functioning as an individual PCR microreactor. Similar to qRT-PCR, total RNA is first reverse-transcribed into cDNA, which is mixed with primers, a fluorescent probe, and PCR reagents before being emulsified into droplets. Following thermal cycling, each droplet is analyzed to determine whether the target sequence was successfully amplified within that droplet. The absolute number of target molecules is calculated using Poisson statistics, bypassing the need for standard curves and making ddPCR advantageous for low-abundance targets or small fold-changes that may fall below the detection limits of qRT-PCR. Because each amplification occurs in isolation, ddPCR is less susceptible to primer–dimer formation or amplification bias caused by reaction kinetics.

RNA-seq leverages high-throughput sequencing to capture the entire transcriptome, without requiring predesigned probes (Wang et al. 2009; McGettigan 2013). Consequently, this method is intended for studying cell-wide impact of experimental conditions of interest rather than to validate, or identify, direct regulators of a GOI. However, as described above, databases collating RNA-seq data can be very useful in predicting potential regulators of a GOI as they allow levels of the GOI transcript to be correlated with other genes, which can be combined with data obtained from open-question experiments that map protein–DNA landscape.

All of the above-listed transcript quantification methods work by isolating RNAs, usually from large cell populations. Consequently, the spatial resolution of gene expression regulation is lost. Spatial resolution can be studied by microscopic imaging techniques. Recent advancements in both staining and imaging technologies have enhanced our ability to visualize gene activity at unprecedented resolution and through various modalities. Although imaging is not generally used to formulate hypotheses about what regulates your GOI, it is indispensable for validating potential regulators in a physiologically relevant context of the cell or organism. Specifically, fluorescence microscopy has emerged as a leading technique for studying gene regulation owing to its unique capabilities that allow researchers to visualize and analyze gene expression in real time at high resolution (Hickey et al. 2021).

Fluorescence microscopy enables the observation of gene expression at the single-cell level, or even single-molecule level with superresolution, providing detailed spatial information about gene activity within cells and tissues. This is crucial for understanding the dynamics of gene regulation during cell growth, differentiation, and responses to stimuli (Zou and Bai 2019). When staining protocols do not require cells to be fixed, fluorescence microscopy imaging is not destructive, allowing researchers to observe live cells without significant perturbation. This is vital when studying dynamic biological processes in real-time, as it minimizes the impact on the system being studied. Techniques such as light-sheet microscopy or spinning disk confocal microscopy further enhance this advantage by reducing photo damage, enabling longer observation times while maintaining high image quality (Eismann et al. 2020). FISH is a powerful imaging-based technique that provides spatial resolution by mapping RNA localization within cells and tissues. It uses fluorescently labeled probes that hybridize to target RNA sequences, enabling detection in a cellular context, down to the level of a single molecule when combined with modern imaging techniques (Young et al. 2020). Despite its limited multiplexing capability owing to the small number of available fluorescence detection channels, imaging is crucial for distinguishing regulatory mechanisms at a GOI that are differentially regulated in different cell types in a complex tissue. A key highlight of FISH is that it provides spatial resolution and the ability to focus on specific transcripts, dissecting transcriptional regulation from potential post-transcriptional regulation mechanisms.

Multiplex hybridization techniques, such as QuantiGene, further enhance RNA detection through a branched DNA (bDNA) system enabling the detection of low-abundance mRNA molecules. Instead of amplifying the target RNA, this system amplifies the detection signal, allowing for the direct quantification of RNA transcripts with exceptional accuracy (Lombardelli et al. 2025). Multiplex hybridization can be followed by quantification of isolated RNA in a tube or in cellulo by a FISH-like approach.

Lately, because of the advances in cellular engineering, it became possible to visualize mRNA molecules in living cells by tagging them with genetically encoded fluorescent RNAs, RNA aptamers that can bind fluorogenic substrates, allowing GOI expression to be monitored in living cells. These aptamers, usually denoted by developers with plant-derived names for easier recognition (Spinach, Broccoli, and Corn in the first generation; Pepper and Clivia in the second), have revolutionized RNA imaging techniques in living cells (Bereiter and Micura 2025). These tools can be used to quantify mRNA levels in individual cells, as well as imaging genomic loci using CRISPR display, real-time tracking of protein–RNA interactions, and superresolution imaging.

Other recent advances include techniques such as proximity ligation of RNA (PLAYR), which allows simultaneous quantification of GOI mRNA and the resulting protein at a single-cell resolution. Unlike other techniques such as RNA-seq and FISH, PLAYR avoids the loss of spatial and contextual information (Duckworth et al. 2019), offering an integrated view of gene and protein expression. PLAYR's compatibility with flow cytometry, mass cytometry, and imaging allows for scalable and high-resolution analysis of regulatory networks in a single assay by using in situ and proximal ligation of target-specific PLAYR probes. These predesigned pairs of hybridization probes bind in close proximity on the transcript of interest. The positioning of the probe pairs creates a platform for the DNA oligonucleotide insert and backbone to anneal. The insert and backbone DNA is then continuously synthesized through rolling circle amplification (RCA) by phi29 polymerase, amplifying the target sequence. Specific detection probes bind to the amplified insert DNA, allowing visualization of transcript-specific PLAYR signal detected via fluorescently labeled oligonucleotides (flow cytometry) or metal tags (mass cytometry) as small punctate dots (Duckworth et al. 2024). Alongside RNA detection, PLAYR also includes antibody staining to visualize GOI protein within cells. Furthermore, designing hybridization pairs to target both spliced and prespliced mRNA expands PLAYR's scope to not only quantify RNA expression but also study RNA dynamics and spatial arrangements during transcription and translation. This spatial and temporal insight into RNA and protein interactions is crucial for understanding GOI regulation in its native cellular context.

However, PLAYR has notable limitations. Although the design of hybridization probe pairs minimizes off-target effects, it increases the overall length of the probes. This poses a challenge for studying small genes, as there may be insufficient space for the required four pairs of hybridization probes to bind effectively (Duckworth et al. 2019). Furthermore, the RCA process introduces potential biases, particularly in signal quantification of expressed genes. These biases may lead to over- or underestimation of RNA abundance, limiting the accuracy in detecting subtle changes. Most importantly, similarly to other methods discussed in this section, PLAYR is an observational method. Although it provides valuable descriptive data, such as RNA and protein localization and abundance, it does not directly establish causation between a regulator and a GOI. To address this, complementary approaches like CRISPR and siRNA libraries need to be used to determine and/ or validate potential regulatory factors of a GOI.

Critically, evaluating changes in transcript abundance upon changes to candidate TF levels using RNA-centric approaches does not clarify whether the TF regulates gene expression directly or indirectly. These approaches also cannot identify the specific regulatory site or the mechanism of action for the TF. To identify the regulators of a GOI, it is crucial to consider all layers of gene regulation. A change in GOI transcript levels can be caused at the level of transcription by TFs and chromatin modifiers. However, GOI transcript levels can also be regulated post-transcriptionally by RNA-binding proteins, as well as microRNAs, and translational modifications or PTMs. Understanding these mechanisms requires tools that can simultaneously capture RNA expression and protein interactions, as these components work together to regulate gene expression dynamically. To gain an understanding of how these factors modulate the GOI, additional experiments discussed later in this article are necessary.

## Experimentally validating TFs by measuring GOI protein product

### Reporter assays

Expression from the GOI can be measured by quantifying the levels of proteins regulated by GOI promoter. To allow for high-throughput analysis, reporter assays integrate GOI promoters with proteins that are easy to detect and quantify such as luciferase. The typical approach utilizes two luciferase reporters, allowing simultaneous measurement of GOI transcription alongside a control, but other types of reporter genes may be used both for the GOI or reference promoter. The most commonly used luciferase enzymes include those from firefly (FLuc or more commonly simply Luc), *Renilla* (RLuc), *Cypridina* (CLuc), *Gaussia* (GLuc), or *Oplophorus* (NanoLuc) (Shifera and Hardin 2010; England et al. 2016). The disadvantage of FLuc and RLuc is the requirement for cell lysis to measure transcriptional activity. Secreted CLuc and GLuc reporters allow cells to remain intact so they can be used in further assays or for long-term monitoring (Wu et al. 2007). NanoLuc is the most versatile system owing to the high sensitivity and low molecular size of the enzyme as well as high stability of the substrate.

Although reporter assays are valuable tools for investigating transcriptional regulation, they have notable limitations. These assays measure the reporter gene's expression controlled by predicted regulatory elements of a GOI. In this artificial expression system, enhancers and/or promoters are inserted into a vector in which DNA methylation is minimal and higher-order DNA structures are absent; consequently, the promoter is always accessible to the transcription machinery. Therefore, although vector-driven reporter assays offer useful insights into how TF could regulate GOI expression (e.g., by using mutated vectors), they do not reflect the complexity of gene regulation in the genomic context. To address this, tags can also be incorporated through knock-in strategies. This allows for endogenous tagging of a GOI, or candidate TFs, a technique that preserves the native regulatory elements. Fluorescent protein (FP) knock-ins can be used for imaging of GOI expression dynamics, observation of TF binding, and chromatin remodeling (Chen et al. 2018; Lionnet and Wu 2021; Viushkov et al. 2022).

Similarly to methods discussed above, luciferase assays simply quantify expression from GOI promoter. Once a list of candidate TF regulators is compiled, we can assess their potential impact on the regulatory element of the GOI through reporter assays. This approach will quantify the effect of a TF on the activation of the GOI promoter and transcription of the reporter gene. However, to evaluate candidate TF impact on GOI expression, reporter assays have to be combined with approaches that alter TF levels. We can cotransfect (1) vectors carrying a control reporter, (2) vectors carrying the experimental reporter, along with (3) a vector encoding the candidate TF. Alternatively, comparison can be made between reporter gene expression in WT cells versus cells that underwent candidate TF depletion.

#### In vivo imaging of reporter genes

In vivo bioluminescence imaging (BLI), just like the typical gene reporter assays described previously, captures light produced by a chemical reaction catalyzed by an enzyme (luciferase) and its substrate (luciferin, which is injected prior to imaging). Unlike fluorescence imaging, BLI does not rely on external light sources, which results in minimal background noise and reduces the risk of phototoxicity. Although the amount of light generated by a bioluminescent reaction is usually too faint to be seen by the naked eye, highly sensitive charge-coupled-device (CCD) cameras can detect and capture this light from tissues or organs in small animals like mice (Keyaerts et al. 2012). In vivo reporter assays use transgenic animals and genome editing techniques, allowing GOI expression reporters to be localized to specific tissues or cellular processes (Yan et al. 2013; Kimura et al. 2014; Serganova and Blasberg 2019). Although technically challenging and costly, this approach provides several advantages over simple cellular models, making it the ultimate choice for validation of GOI regulators (Kvon 2015). Transgenic animal models maintain the complexity of whole organisms, including tissue architecture, systemic responses, and response to the environmental influences. This allows us to study GOI expression regulation in the context of natural biological conditions (Li et al. 2018b). Moreover, in vivo reporter assays enable the assessment of GOI activity across various tissues and organs simultaneously, which is particularly useful to understand how GOI is regulated under different physiological conditions (Maguire et al. 2013). Finally, because reporters used for in vivo studies are designed to be imaged by noninvasive techniques, changes in gene expression can be monitored dynamically over time (Buckley et al. 2015; He et al. 2019).

Detecting bioluminescence beyond a few centimeters remains challenging, as light signals must travel through tissue that absorbs, attenuates, and scatters their emissions (Curtis et al. 2011). Another common shortcoming of BLI assays is the hardship of standardization. The signal depends on many components like the concentration and stability/availability of luciferin, luciferase, and cofactors. Factors like oxygen levels, pH, temperature, and the choice of substrate and administration method affect the BLI signal in vivo. Standardization and validation are crucial for accurate data interpretation, and continuous technological advancements help in overcoming some of these challenges (Badr 2014). As BLI becomes more widely used in biological research, new luciferase genes and their mutant variants are being developed, alongside structural modifications to both the luciferases and their substrates (Xu et al. 2016; Mezzanotte et al. 2017; Saito-Moriya et al. 2021). The aim is to increase photon emission, expand the range of emission wavelengths for multispectral imaging, and shift emissions to longer wavelengths (>620 nm) to improve tissue penetration in small-animal models.

Alternatively to BLI, in vivo fluorescent imaging (FLI) can be used. It utilizes FPs, probes or ligands, which emit light upon excitation by an external light source. FLI stands out among other imaging methods owing to its minimal invasiveness, ability for real-time and multiplexed imaging, and relatively low cost. Because of the high number of photons generated, FLI typically requires very low concentrations of fluorophores (pico- to femtomolar) to produce high-contrast images, which helps lower both the cost of probe production and the risk of toxicity (Refaat et al. 2022). However, autofluorescence from tissues can interfere with the signal, reducing both contrast and accuracy. Additionally, photobleaching can occur with prolonged light exposure, causing fluorescent molecules to lose their ability to emit light and compromising image quality over time, which limits the potential for long-term kinetic studies. Furthermore, similarly to BLI, the main limitation of FLI is tissue penetration, as photon absorption and scattering reduce visible light intensity by about 10-fold for every centimeter of tissue (Sadikot and Blackwell 2005). To overcome this, advances have been made to develop reporters emitting in near-infrared (NIR) (Richie et al. 2017; Ding et al. 2018). At NIR wavelengths (650–1700 nm), light is absorbed and scattered by biological tissues to a lesser extent, whereas background autofluorescence decreases and is virtually nonexistent in the 1500- to 1700-nm spectral band (Diao et al. 2015).

In addition to the most commonly used BLI and FLI reporters, deep-tissue reporters have been developed to make use of medical imaging techniques. Some efforts have also been made to develop reporters perceptible with techniques better suited for bigger animals. These include PET reporters (Nerella et al. 2023), SPECT reporters (Wu et al. 2013), and MRI contrast reporters (Cheng et al. 2017; Concilio et al. 2021). Among various types of reporter genes, each has its own set of advantages and limitations depending on the imaging technique used. Fused reporter genes or single reporter genes detectable by multiple modalities can help overcome drawbacks while enhancing the benefits of each imaging method (Li et al. 2018a). These multimodal reporter genes include dual FP reporters (Camino et al. 2020), dual fluorescence and luminescence reporters (Choi et al. 2016), dual luminescence and radionuclide reporters (Gaspar et al. 2024), dual luminescence and MRI reporters (Rossi et al. 2016), or dual radionuclide and MRI reporters (Patrick et al. 2014).

### Functional assays

Unlike gene reporter assays, which often involve introducing “naked” DNA constructs with reporter genes under control of artificial promoters, functional assays assess gene regulation within the natural chromatin environment. As a result, functional assays can provide an understanding of how gene regulation impacts biological processes, offering insights that are closely aligned with the gene's natural regulatory mechanisms. By combining these assays with tools like CRISPR-Cas9 or rapid protein depletion, researchers can directly manipulate gene expression, observing how these changes influence protein function, cell behavior, or organismal traits in a context that mirrors biological conditions. These insights are invaluable for understanding the mechanisms of gene regulation and for validating computational data experimentally.

One key challenge of functional assays is that they measure the outcome of gene regulation, such as protein levels, enzyme activity, or cellular behavior, without identifying the specific regulatory level at which changes occur. For example, an increase in protein expression observed through a functional assay might be a result of enhanced transcription, but it is just as likely to be caused by increased mRNA stability or by more efficient translation; however, the assay itself will not distinguish between these possibilities. This ambiguity makes it impossible to determine if observed changes are caused by direct regulation of GOI expression.

#### Measuring the protein product

One indirect way of assessing changes in GOI expression is by measuring the levels of protein product encoded by the GOI upon changes in candidate TF levels. Assays that measure protein quantity and /or activity include western blotting, ELISA, enzyme activity assays, and receptor-binding assays. For example, ELISA quantifies protein expression by detecting specific antigens. Enzyme activity assays indirectly assess how gene regulation impacts the amount of enzyme capable of catalytic function (Vimalraj 2020); however, this relies on a simplistic assumption that there is a direct correlation between the amount of the enzyme and its activity level, which is not always true, for example, when excess inhibitors might be present. Similarly, receptor binding assays examine if GOI-regulated receptor expression is changed upon TF increase/decrease. These assays collectively offer a “quick and dirty” method to validate candidate TFs, although it is critical to remember that changes in protein quantity or activity might not be a direct result of GOI expression. It is just as likely that processes such as splicing, translation, protein stability, PTM, or chaperone levels have been affected causing a change in the readout levels.

#### Measuring GOI protein levels using fluorescent tagging

Tagging the GOI with FPs is another method to track GOI expression, as well as to follow TF-driven regulatory events. Time-lapse imaging enhances this approach by enabling collection of data over extended periods, facilitating comprehensive analysis of gene expression luminesce fluctuations (Liu and Tjian 2018). In the past two decades hundreds of new FPs covering the visible light spectrum have been engineered and become indispensable for the visualization and quantification of gene expression (Specht et al. 2017; Lukyanov 2022). Moreover, many new variants of FPs (photoactivatable, photoswitchable, reversibly photoswitchable) use advanced imaging techniques and allow time-dependent analysis and real-time tracking of transcriptional processes in single cells (Wu et al. 2011). As a result, a variety of computational techniques have emerged to quantitatively reconstruct promoter dynamics from time-lapse measurements (Lichten et al. 2014; Zulkower et al. 2015; Kannan et al. 2018), lately even taking into account FP maturation dynamics (Pavlou et al. 2022). Another way of taking advantage of distinct kinetics of fluorophore maturation in FPs is the use of tandem FP timers (tFTs), which are versatile reporters of protein dynamics (Khmelinskii and Knop 2014). When combined with high-throughput genome editing, tFTs can be used to identify regulators of GOI that differently impact the rate of protein synthesis, maturation, and degradation (Fung et al. 2022).

#### Cell-based assays

Cell-based assays can also be used as a “quick and dirty” way to validate the impact of candidate TF on the GOI expression, providing that GOI is a regulator of cell division, cell death, metabolism, or another easy-to-measure process. For GOIs that regulate cellular proliferation, assays such as colony formation, cell counting, or cell cycle profiling can be used upon candidate TF level manipulation. Similarly, migration assays, including chamber-based methods, evaluate cellular motility and can be used to validate TFs of GOIs that control these processes. By integrating cell-based assays with other molecular methods like reporter assays, researchers can try to define the effect of candidate TFs on GOIs in a quick, technically easy, and cost-effective way.

To summarize, in vitro and in vivo reporter assays as well as fluorescent tagging of endogenous GOI loci or GOI product detection allow us to quantitate expression of the GOI; however, these methods alone are not able to identify, or even validate, GOI regulators. Perturbation of candidate TF levels is required to make these quantitative observations informative.

## Experimentally validating if candidate TF binds to the GOI

To identify direct regulators of GOI expression, it is critical to confirm their ability to bind to the GOI. Usually a candidate TF regulator of a GOI is identified in silico first, but this relationship has to be validated by confirming the binding between the TF and the GOI regulator DNA sequence. Methods outlined in this section targeted to specific proteins work well for validating regulators of the gene in question, rather than for the discovery of novel GOI regulators, as they rely on study-specific fusion proteins.

The basic method for identifying DNA–protein binding in the molecular geneticist's repertoire has already been mentioned: supershift EMSA, which analyses the capacity of individual TFs extracted from cells’ nuclear lysate to bind to a labeled DNA probe. EMSA's fundamental limitation is the reliance on naked DNA, lacking all chromatin context. To overcome this, experiments can be coupled with targeted ChIP, which is currently the entry-level golden standard in supporting direct TF-GOI binding (Kim and Dekker 2018). Chromatin is reversibly cross-linked with formaldehyde, sheared, and immunoprecipitated with antibodies against the TF to be tested. The precipitate is de-cross-linked, and the abundance of DNA derived from regulatory sequences that were bound to the TF in question, as a measure of its relative occupancy of the response elements in the cell population, is assayed. TF-bound DNA can be quantified by simple qPCR, with nonregulatory sequences known not to bind the TF, or regulatory sequences in which the binding of investigated TF is known not to be influenced by the treatment in question, used for reference. The main drawbacks of this technique are the need for large cell numbers, as well as artifacts related to antibody quality, chromatin fixation, and shearing, combined with risk of false-negative results when an incorrect location in the gene promoter/enhancer is selected for detection.

Alternatively, DNase I footprinting offers a classical biochemical approach to map exact protein–DNA interaction sites at base pair resolution. Radiolabeled or fluorescently labeled DNA is incubated with a purified DNA-binding protein or nuclear extract and then subjected to limited digestion by DNase I. Regions of the DNA protected from cleavage by bound proteins are visualized as “footprints” compared with a control digestion in the absence of protein. This method provides detailed structural information about binding sites and can distinguish between specific and nonspecific DNA–protein interactions. However, it is technically demanding and low-throughput and requires relatively large amounts of purified protein and clean DNA, limiting its use.

When reliable and quantitative data are a priority, rather than the cellular context, binding between the candidate TF and GOI regulatory sequence can be assayed by direct biophysical techniques. These approached require high-quality purified TFs but give definite answers on a TF's absolute ability to bind a response element and the kinetics of such binding. Although spectroscopic techniques detecting conformation change, such as circular dichroism, have been successfully applied for this task, the current technique of choice is surface plasmon resonance (SPR), in which one interacting partner (DNA or protein) is immobilized on the surface of a flow cell, and this surface is kinetically exposed to a solution of the other partner. Upon binding, the average molecular mass of complexes at the surface increases, changing the properties of plasmons (energy vibrations induced in interface matter by light upon total internal reflection) and allowing optical detection by monitoring the attenuated total internal reflection angle. This powerful technique has been successfully applied to detect weak, transient TF–DNA interactions or binding facilitated by other interaction partners (e.g., coactivators) (Moyroud et al. 2009; Aditham et al. 2021).

### Microscopic imaging of TF–GOI interactions

When identification of physiologically occurring TF–GOI interaction is of paramount importance, imaging techniques are the go-to approach (Lee and Myong 2021). Their basic application can be divided to simple colocalization or proximity based. For colocalization analysis, binding partners, which are usually the candidate TF and other proteins already known to bind to the characterized GOI promoter sequence (stained, e.g., by DNA-FISH), are fluorescently labeled (e.g., by immunofluorescence) and visualized at highest possible resolution to determine the proportion of pixels when both co-occur. Similarly to functional assays, these simple interaction-detection approaches are an easy and cheap way of verifying GOI candidate regulators (Chaumeil et al. 2013; Gilbonio et al. 2023). A positive result does not confirm binding but is a strong indication of a possible interaction especially when superresolution microscopy is employed (Dai and Dai 2012). Unfortunately, these techniques suffer from drawbacks including poor signal-to-background ratio and limited specificity and spatial resolution. Furthermore, because high-throughput widefield fluorescence microscopy images cannot resolve crowded molecular targets, the amount of extractable biological information is often limited. One way to overcome some of these limitations is to apply advanced computational methodologies of image deconvolution (Wernersson et al. 2024). However, simply localizing DNA and protein in one place is not enough to prove regulatory mechanisms as elegantly outlined by the Hahn group (Mahendrawada et al. 2025). To quantify DNA–TF interactions based on molecular distance Foerster resonance energy transfer (FRET) between two fluorophore molecules attached to interacting partners, DNA–TF, can be detected by fluorescence microscopy (Chen et al. 2021b; Wang et al. 2022). Alternatively, in bioluminescence resonance energy transfer (BRET), the energy donor is not a fluorophore but a light-emitting enzyme, for example, luciferase (Vickers and Crooke 2016).

### High-resolution imaging of TF–GOI interactions

Superresolution fluorescence microscopy (SRM) technologies developed over the past two decades have pushed the resolution limit of fluorescently labeled molecules into the nanometer range and revolutionized our understanding of gene regulation by providing unprecedented insights into the spatial organization and dynamics of chromatin and transcriptional machinery at the nanoscale (Xu et al. 2020; Parteka-Tojek et al. 2022). These advanced imaging techniques enhance resolution by refining illumination and detection strategies, including methods like stimulated emission depletion (STED) microscopy, structured illumination microscopy (SIM), and single-molecule localization microscopy (SMLM) approaches such as PALM and STORM (Birk 2019). The ability to directly detect individual fluorescent molecules in living cells enhanced our comprehension of how gene expression is regulated within the cellular environment and with close-to-physiological cellular context (Uphoff 2016). High-throughput superresolution imaging can now reveal 3D chromatin organization, resolving prior uncertainties, challenging existing models of regulatory specificity, and suggesting new roles for chromatin structure in transcription (Brandão et al. 2021; Hafner and Boettiger 2023). Although SRM techniques usually rely on fluorescent labeling of proteins of interest (like TFs), an interesting alternative is inverse imaging of unlabeled proteins bound to DNA. This technique relies on DNA-binding fluorophores that transiently label bare but not protein-bound DNA, making it possible to observe unlabeled protein patches bound to isolated strands of DNA with a resolution limit estimated between 5 and 15 nm in size (Meijering et al. 2020). Despite its powerful capabilities, SRM requires expensive and time-consuming sample preparation, robust imaging protocols, and access to advanced microscopic equipment and software to ensure accurate and reproducible results (Chen et al. 2024a).

Alternatively, expansion microscopy (ExM) enhances the resolution of light microscopy by physically enlarging biological samples, allowing for detailed studies of gene regulation at the nanoscale. ExM employs a hydrogel embedding process in which fixed and immunolabeled cells are treated to swell isotropically, effectively increasing the distance between labeled molecules. This expansion allows conventional microscopes to resolve structures that were previously below the diffraction limit of light microscopy, achieving lateral resolutions of ∼70–80 nm (Wen et al. 2023).

ExM has been used to map epigenetic interactions (Acke et al. 2022), investigate the nanoscale organization of chromatin during transcription (Pownall et al. 2023), analyze nuclear structures in cultured cells, and facilitate the examination of spatial relationships between proteins involved in gene regulation at a subnuclear scale (Holsapple et al. 2023; Mäntylä et al. 2023). This allowed researchers to observe interactions between different nuclear components and their roles in gene expression (Faulkner et al. 2022). Although ExM provides remarkable insights, it also presents significant challenges. The process can be time-consuming and requires careful optimization of hydrogel chemistry and sample preparation to ensure isotropic expansion and minimize artifacts. Variations in expansion factors across different cellular structures can complicate data interpretation, necessitating rigorous controls and validation (Gaudreau-Lapierre et al. 2021).

In summary, although useful in many cases, one major drawback of using visualization-based approaches is that they require prior sample fixation and thus necessitate mindful planning of experimental data points. Furthermore, as discussed above, chromatin accessibility and DNA–protein colocalization are simply prerequisites for binding and expression regulation.

### DNA adenine methyltransferase identification

**Figure 2.**
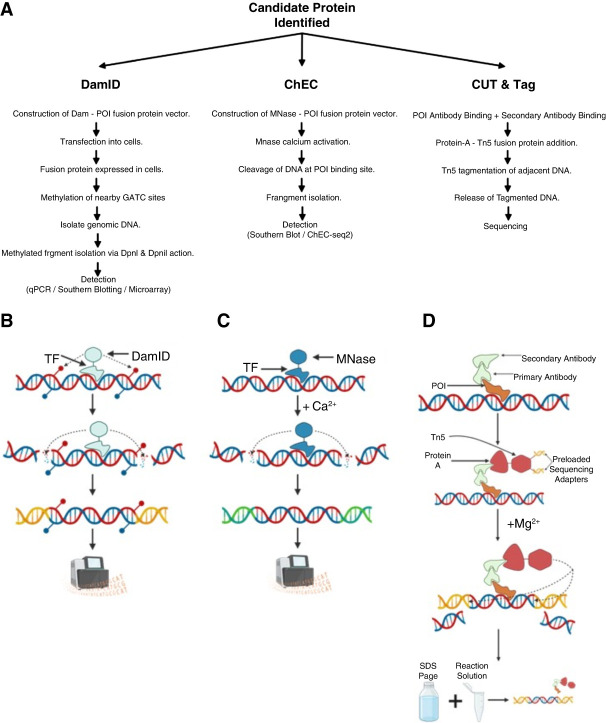
Comparison between select protein focused protein–DNA interaction analysis methods. (*A*) DamID (Greil et al. 2006; Aughey and Southall 2016), ChEC (Schmid et al. 2004), and CUT & Tag (Kaya-Okur et al. 2019) workflows following candidate protein identification. (*B*) DamID-sequencing. DamID-fusion with candidate TF binds to DNA and proceeds to methylate nearby adenines at the N6 position within GTAC motifs. Following DNA isolation, DpnI selectively cleaves DNA at GmATC positions. Cleaved DNA is ligated to primers for PCR amplification. After sufficient amplification, DNA is sent for sequencing. (*C*) ChEC-sequencing. MNase-candidate TF fusion binds to DNA. Cells are permeabilized, and Ca^2+^ is added, causing MNase to cleave nearby DNA. Cleaved DNA is isolated and ligated to primers for PCR amplification. After sufficient amplification, DNA is sent for sequencing. (*D*) CUT&Tag. Cells are isolated and permeabilized, with primary antibody added to bind to target protein and secondary antibody added to increase yields. A Protein-A–Tn5 fusion protein is added, binding to the secondary antibody. Mg^2+^ is added to activate the Tn5. Tn5 cleaves adjacent DNA and ligates sequencing adapters to DNA. SDS-PAGE is added to the reaction to release DNA fragments, ready for sequencing.

DNA adenine methylase identification (DamID) can be used to validate, or identify, the DNA binding sites of candidate proteins and can be seen as an alternative to ChIP (Fig. 2A,B). A fusion product made up of the candidate TF and an *Escherichia coli* DNA adenine methyltransferase (Dam) is generated. When candidate TF binds to the DNA, the attached Dam methylates nearby adenines at their N6 positions in dsDNA within GATC sequences (Barras and Marinus 1989). Because adenine methylation does not occur naturally in most eukaryotes, this provides a unique tagging system, marking the binding sites of candidate TF. After expression of the Dam fusion protein, methylated adenine regions are cleaved by DpnI, which selectively recognizes G^m^ATC sites (van Steensel and Henikoff 2000), and amplified using a methyl-specific PCR reaction (Greil et al. 2003). Amplified fragments of DNA that were bound by candidate TF can be detected using qPCR, Southern blotting (Greil et al. 2006), or DamID-seq (Wu et al. 2016). Because DamID uses cloning resulting in expression of the fusion protein inside a living cell, no cross-linking is required, reducing the risk of artifacts.

Because of methylation-based detection, DamID lacks precision; the spread of methylation may be broader than the binding site, and unbound fusion protein will inevitably diffuse through the nucleus and, because of Dam's high affinity for GATC sequences, produce considerable levels of background methylation (Greil et al. 2006). Another limitation is that chromatin structure can restrict access of the Dam–TF fusion protein to certain regions of the DNA, leading to potentially incomplete or biased mapping of binding sites; however, this improves biological relevance (Gottschling 1992). A further drawback of this method is that it is dependent on the availability, as well as the frequency, of GATC sequences within the GOI; Dam methylation can occur up to ∼5 kb from a binding site (Greil et al. 2006). However, by generating a fusion protein of candidate TF and comparing it to nonspecific Dam fusion, it allows experimental validation of TF binding within the GOI regulatory sequences. It is important to point out that candidate protein is introduced artificially as a fusion. Furthermore, just like ChIP-based methods, DamID characterizes DNA binding profile of a TF of interest and hence cannot help in predicting regulators of a GOI if these are not known.

### Chromatin endogenous cleavage

Chromatin endogenous cleavage (ChEC) (Schmid et al. 2004) is another ChIP alternative; it involves generating a fusion protein, consisting of the candidate TF and a C-terminal micrococcal calcium–dependent endonuclease (MNase) (Fig. 2C). When candidate TF binds to its target sites on the DNA, MNase is activated through the artificial increase of calcium ion concentration. Once activated, MNase cleaves DNA nearby to the binding site of the candidate TF (Babl et al. 2015). Cleaved DNA fragments can be identified at specific, single loci, through the use of Southern blotting and indirect end-labeling (Schmid et al. 2004), although these methods have been surpassed by newer developments. More recently, ChEC-seq has been developed to incorporate high-throughput sequencing (Zentner et al. 2015), DNA fragments are isolated through negative selection using size-selecting magnetic beads; DNA ends are repaired and subsequently ligated to Illumina TruSeq adapters for sequencing. Additionally, ChEC can be used to provide data on the frequency of the protein–DNA interactions; the degree of DNA degradation by the MNase fusion protein can be monitored in time course experiments (Merz et al. 2008). Similarly to DamID, thanks to the production of a fusion protein, cross-linking is not required, reducing the risk of artifacts.

However, when highly abundant proteins are studied, MNase has the potential to produce excessive cleavage, which makes it more challenging to confirm specific binding events. It is important to consider that observed cleavage may not represent direct binding of the protein to specific DNA sequences, as some cleavage may occur owing to spatial proximity rather than direct interactions. However, background signal caused by nonspecific cleavage can be addressed through the recently developed bioinformatic filtering, DoubleChEC, which produces robust identification of TF binding motifs and target genes (VanBelzen et al. 2024). Another drawback of the original ChEC-seq is that proteins that sparsely interact with DNA yield relatively few DNA fragments. However, this has been addressed in ChEC-seq2 (VanBelzen et al. 2024), in which free ends of the genomic fragments are marked through ligation to a custom adapter, which is followed by Tn5 transposase-mediated library construction. These fragments are then specifically amplified with the Nextera index primers, resulting in a DNA library compatible with Illumina sequencing. Similarly to DamID, a candidate protein is introduced artificially as a fusion.

### Cleavage under targets and tagmentation

Cleavage under targets and tagmentation (CUT&Tag) is based upon the same immunotethering principles as ChIP, which are described above (Kaya-Okur et al. 2019). However, in CUT&Tag, after antibody incubation, instead of immunoprecipitation, chromatin shearing and library preparation are performed (Fig. 2D).

First, cells are isolated and immobilized on magnetic beads, permeabilized, and treated with an antibody-specific to the candidate TF, along with a secondary antibody to amplify the assay yields. A fusion protein made up of Protein A–Tn5 (transposase), which binds to the antibody-labeled chromatin, is added to the reaction. The Tn5 comes preloaded with sequencing adapters for high-throughput sequencing. Mg^2+^ is added to the reaction to activate Tn5, which cleaves the adjacent chromatin and ligates the sequencing adapters to the DNA. SDS buffer is added to the solution, releasing the tagmented DNA and allowing for it to be sequenced (Kaya-Okur et al. 2019).

CUT&TAG is simpler and cheaper than ChIP-seq; all the steps are compatible with a single test tube workflow and take just 2 days. Costs are lower because there is no separate library preparation step (Kaya-Okur et al. 2019; Henikoff et al. 2020). Critically, CUT&Tag works with very low cell numbers and even single cells (Kaya-Okur et al. 2019).

However, CUT&Tag can give high background owing to Tn5 binding open chromatin and/or mitochondrial DNA. Nonspecific noise caused by mitochondrial DNA binding can be avoided by using nuclei rather than whole cells. CUT&Tag requires high-salt washes to remove nonspecific or unbound fusion-protein before tagmentation to help reduce background noise; however, these high-salt washes may impact weaker chromatin interactions, potentially affecting biological relevance of the data when mapping proteins that do not bind to the DNA as strongly. It is important to consider that, similarly to ChIP-seq, DamID, ChEC, and CUT&Tag are much better suited for identifying genes bound to a protein of interest rather than for validating candidate protein binding to a GOI. These methods involve significant bioinformatic analysis of a large number of sequences identified for each protein of interest, and this richness of information is simply not needed to confirm if protein X binds to a single GOI (Fig. 2A; Table 1).

**Table 1.** Comparisons of ChIP, DamID, ChEC, and CUT&Tag

Aspect of the method	ChIP	DamID	ChEC	CUT&Tag
**Principle**	Antibody used to precipitate protein of interest with the DNA it is bound to, which is resolved by DNA-seq.	Fused DNA-binding protein with DAM methylates adenines at binding sites, detected via sequencing.	Uses MNase-fused DNA-binding protein to cleave chromatin at DNA-binding sites.	Uses protein specific antibodies in conjunction with Tn5 transposase to tag and sequence protein-bound DNA.
**Strengths**	Genome-wide mapping of protein–DNA interactions with high resolution. Well-established, standardized protocols and widely supported bioinformatic pipelines.	Direct mapping of protein–DNA interactions within cells. No cross-linking required; avoiding artifacts. Does not require the use of antibodies.	Produces robust data when used in conjunction with DoubleChEC. Captures transient interactions. Does not require cross-linking; avoiding artifacts.	High-resolution mapping. Works effectively with a relatively small number of cells. Reduced sequencing depth compared with other immunotethering-based methods.
**Limitations**	Relies on the presence of highly specific antibodies. Requires cross-linking, which can cause artifacts. Will not capture transient interactions. Antibody binding can skew the results.	Limited by chromatin structure. Can produce unintended methylation, leading to background signals. Requires introduction of fusion protein, which is not at a physiological level.	Less suitable for work on highly abundant proteins. Requires cell permeabilization. Requires introduction of fusion protein, which is not at a physiological level.	Relies on the presence of highly specific antibodies. Nonspecific noise may be caused by mitochondrial DNA. Potential for missing data related to low-affinity DNA-binding proteins.
**Specificity**	As specific as the antibody.	Moderately specific; potential for bias owing to the preferential methylation of adenine in specific sequence contexts.	Highly specific for protein binding sites, enhanced by the ability to control activation of MNase.	As specific as the antibody.

When assessing whether a TF regulates a GOI using data sets, genome-wide data offer key advantages over single-locus measurements. Techniques such as ChIP-qPCR or reporter assays can provide sensitive, targeted information, but they are inherently limited by primer design, locus selection, and assumptions in enrichment calculations. In contrast, genome-wide approaches such as ChIP-seq, DamID-seq, ChEC-seq CUT&RUN, or CUT&Tag generate comprehensive profiles of TF binding that can be rigorously normalized and benchmarked across positive and negative controls. These data sets also allow for the discovery of enriched DNA motifs and cobinding partners, providing broader biological context that single measurements cannot capture. Importantly, genome-wide assays make it possible to distinguish genuine TF binding events from technical artifacts and to evaluate binding specificity across the genome. Thus, although locus-specific assays remain useful for validation, genome-wide strategies provide a more robust and controlled framework for inferring direct TF–gene regulatory relationships.

In parallel with experimental approaches, computational prediction of TF targets using deep learning is rapidly gaining traction (Choi et al. 2025). Models can infer regulatory relationships directly from DNA sequence, epigenomic features, or large-scale perturbation data sets such as Perturb-seq (Dixit et al. 2016), offering the potential to nominate candidate TF–gene interactions at scale. Although these predictions are powerful, they should be interpreted cautiously: Model accuracy depends on training data quality, and predicted interactions require experimental validation. With the rise of large language models (LLMs) and other AI approaches, the use of in silico predictions is likely to proliferate, but they should complement, not replace, empirical measurements when establishing causal TF–gene relationships.

### How to make an ideal cell-based reporter assay

**Figure 3.**
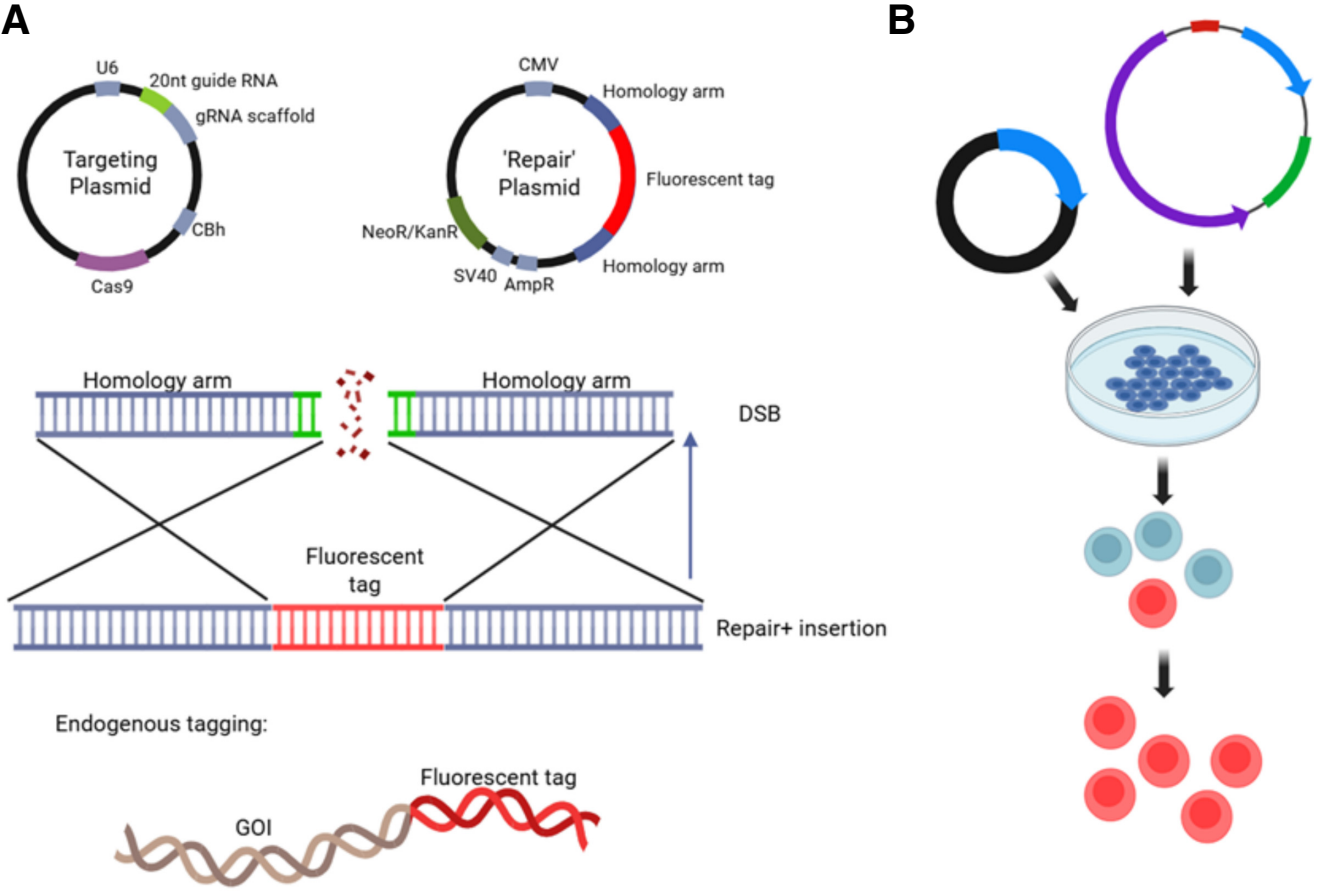
Generating a reporter cell line of the endogenous locus. (*A*) Using CRISPR-Cas9 to tag a gene of interest (GOI) with a fluorescent tag at the C or N terminus. (*B*) Following transfection of the CRISPR-Cas9 plasmids and selection of the tagged GOI cell line, GOI expression from its endogenous locus can be quantified by measuring fluorescent signal.

An ideal gene regulation assay consists of two components: an effective and well-researched TF or treatment and a reliable, consistent detection method. In the simplest terms, the yeast one-hybrid (Y1H) assay provides a genetic approach to identify TFs that bind to a specific DNA sequence in vivo. The DNA bait, GOI promoter, is integrated into the yeast genome upstream of a reporter gene. Yeast is then transformed with libraries expressing potential TFs fused to a transcriptional activation domain. Binding of a factor to the bait DNA drives reporter expression, revealing functional interactions. Y1H is especially useful for screening large cDNA libraries to uncover novel regulators. However, its utility is constrained by the heterologous yeast system, which may not recapitulate all aspects of human chromatin structure or protein folding, and by the possibility that certain interactions require cofactors absent in yeast. Additionally, as with all artificial systems, false positives and negatives are a known limitation, necessitating downstream validation in the native cellular context. However, this approach can be improved through the use of CRISPR-Cas9 technology, which enables the incorporation of gene reporters directly into the human genome. By knock-in, a reporter tag can be attached to a specific gene and expressed stably, thus creating a cell line with a permanent reporter expression (Fig. 3A; Damhofer et al. 2021; Tamura and Kamiyama 2023; Deleuze et al. 2024). Contrary to traditional plasmid systems, CRISPR knock-in preserves a GOI's native promoter, enhancers, and chromatin context. Real-time changes in GOI expression can be observed in response to stimuli, TFs or chromatin modifications, directly identifying key regulators.

This method has been commonly used with a NanoLuc reporter incorporated into the genome, allowing for luciferase-based quantification of expression (Oh-Hashi et al. 2016; Li et al. 2019a). Similarly, FPs can be used, allowing product visualization in a range of cell lines, including brain neurons, hiPSCs, and HeLas (Uemura et al. 2016; Koch et al. 2018; Sharma et al. 2018). Incorporation of a fluorescent tag enables efficient and effective fluorescence-activated cell sorting (FACS), allowing for isolation of distinct regulatory states for further analysis with RNA-seq or ChIP-seq.

Once generated, a stably tagged knock-in cell line is a reliable cell-based reporter that can be assayed on a large scale (Fig. 3B). However, creation of the cell line poses several challenges. Consideration must be made for which end of the protein is tagged (predictive web tools can determine if functional regions or folding will be perturbed) (Käll et al. 2004; Roy et al. 2010), the importance of subcellular localization and trafficking (Teufel et al. 2022), and the assurance of minimal disruption of protein interactions (Omasits et al. 2014), as these are critical for accurate detection at protein level. Alternatively, a promoter can be tagged with a fluorescent sequence downstream; this answers if the promoter is activated but the FP is produced on its own, without compromising the target product (Ijaz and Ikegami 2021). This approach uncouples gene expression from transcription, allowing separate quantification of translational and transcriptional activity in a single system by tagging both the promoter and target gene with differing tags. However, tagging the promoter is challenging as some promoters have multiple regulatory elements; this makes tagging the GOI easier to interpret in the context of its natural regulation.

Another consideration is what to tag with. Fluorescent tags are relatively bigger than other knock-in options and so can pose challenges experimentally. Smaller tags such as mNeonGreen and mScarlet, which are compact but bright FPs, may prove easier to both insert and detect (Shaner et al. 2013; Bindels et al. 2017). However, these are newer and less tested with knock-in applications (Crowe and Yue 2019; Morrow et al. 2021). Both eGFP and mCherry are more widely used, photostable options with established knock-in protocols (Lee et al. 2022; Kim et al. 2023, 2024a; Tamura and Kamiyama 2023; Jiang et al. 2024). If you want to tag multiple genes in one system, mCherry and eGFP are also an excellent combination for flow cytometric analysis (Kleeman et al. 2018). Further gene tagging requires a FACS machine capable of blue excitation; for this, mTagBFP pairs well (Subach et al. 2011; Perez-Leal et al. 2021). Lastly, as with most CRISPR-Cas9 experiments, care must be taken to screen for unwanted mutations and produce a homozygous cell line to reduce noise from expression level differences (Koch et al. 2018; Hunt et al. 2023).

Users must consider the time and care needed to create a stable knock-in cell line for a GOI and balance this against its advantages, including the preserved chromatin context and the dependable nature of the system for long-term investigations. The time-consuming nature of producing a cell-based reporter line can be mitigated by using the latest advances in CRISPR knock-in techniques. One example is the “HiTag” technique, which can incorporate a tag into primary cells, which are difficult to clonally select, as well as postmitotic cells, with a single electroporation step (Zeng et al. 2020). This method has been used successfully with mCherry to produce a C-terminal tagged protein library (Kim et al. 2024b). Another method is targeted knock-in with templates (TKIT); this approach uses two-guide RNA and donor templates (Fang et al. 2021). TKIT was used successfully to tag synaptic proteins with mNeonGreen in mouse cortical neurons.

Successful creation of a homozygous tagged cell line offers a method for systematically identifying what regulates a GOI. The endogenous reporter for a GOI line can be treated with specific TFs through plasmid delivery or direct protein addition (Del'Guidice et al. 2018) as a direct measure of gene activation or repression. Further, cell populations or individual cells can be sorted based on fluorescence levels using FACS and kept for downstream applications such as RNA-seq (Grolmusz et al. 2016) or techniques described in the previous sections, imaging using the cells genetically encoded fluorescence, further flow cytometry for additional markers, ChIP-seq, and western blot. CRISPR knock-in can also be combined with the already established CRISPR knockout libraries to identify GOI regulators (Sanson et al. 2018). Through introduction of knockouts in predicted regulatory regions, fluorescence changes representing altered GOI expression can be quantified, revealing factors that control GOI expression. This high-throughput technique could reveal upstream regulators, pathways, and chromatin modifications involved in GOI control.

Using a CRISPR-Cas9 knock-in tag to determine what regulates a GOI offers a powerful approach that has significant advantages over traditional methods. This technique provides a permanent biosensor integrated into the genome at one time point, allowing for consistent long-term expression without the need to incorporate a detection method at each assay, thus increasing experimental reproducibility and reducing technical inconsistencies.

### Summary

Targeted protein centric methods that analyze known candidate protein binding to the DNA using fusion proteins or immunodetection, although powerful for identifying DNA bound to a protein of interest, are not good tools for validating candidate proteins suspected of binding to a GOI. Generating fusion proteins with the TF of interest is often required, and the process can be resource-intensive when dealing with a large candidate list. Therefore, prioritizing candidates through complementary approaches or bioinformatics can streamline the validation process. Furthermore, it is critical to remember that not all of these methods explore if candidate protein binding has any effect on GOI expression, and as such, they cannot confirm a regulatory role. Similarly, methods based on visualizing chromatin or DNA–protein colocalization give no insight into a regulatory role and need to be coupled with other assays to identify, or even validate, candidate TFs of a GOI. However, recent developments in genome editing allow for smart and ambitious protocols to be implemented.

## Identification of proteins bound to the regulatory elements of the GOI within the cell

To discover what regulates the expression of a GOI, we need to be able to map which proteins bind to its regulatory elements on the DNA. There has been a lot of effort to address this challenge, resulting in reverse ChIP and in proteomics of isolated chromatin segments (PICh) development. Both of these methods leverage MS/MS analysis to identify proteins bound to the DNA of interest; however, reverse ChIP does this in vitro, whereas PICh allows for cell-based analysis. The principle is that if the expression of a GOI is increased when cells are subjected to condition X, by comparing which proteins are bound to the GOI regulatory element upon treatment X, in contrast to an untreated control, we can determine proteins that drive GOI expression, at least in that condition. This offers a significant step up from methods that rely on artificially introduced fusion proteins and, as such, are not suited for testing if candidate protein binding to TF affects GOI expression.

### Reverse chromatin immunoprecipitation

For both reverse ChIP (r-ChIP), also known as the DNA pull-down assay (Wen et al. 2020; Wen and Wang 2024), and PICh (Déjardin and Kingston 2009), the DNA sequence of interest, such as the GOI promoter or enhancer, needs to be determined first as described above. Once this sequence is known in reverse ChIP, the sequence needs to be synthesized with tags that will allow pull-down such as biotin. Cells are grown under conditions of interest followed by lysis and fractionation to isolate the DNA-bound proteins. Chromatin fraction undergoes sonication to shear the DNA, leaving the proteins that were bound to the DNA. The resulting protein solution is incubated with the synthesized GOI DNA–biotin construct. Following washes and pull-down, or magnetic bead enrichment, proteins that are specifically bound to the DNA of interest are isolated and can be identified using MS/MS. However, there are few set backs to this approach. Controls need to be carefully designed, including scrambled DNA sequence, beads alone, etc., to weed out nonspecific binders. Construct length needs to be carefully considered, taking into account binding motifs as well as specificity. Furthermore, careful consideration needs to be made when deciding which end of the DNA should be tagged as that might affect protein binding. Similarly, there can be an advantage in cross-linking protein–protein complexes to ensure their competency to bind the DNA; however, increased binding can significantly increase the amount of nonspecific hits. The most important complication arises from the design of this experiment in which proteins are exposed to, in essence, a dsDNA break. Consequently, there is recruitment of DNA ds break repair machinery, which is not specific to the GOI sequence and competes with specific binders. Of course, the use of scrambled DNA as a control is meant to combat this, but in practice, this is a major obstacle. Some studies have attempted to further enhance specificity by using probes with single-base mutations at sites known to play a critical role for TF binding; however, if this level of knowledge about the GOI, and its TF binding, already exists, the whole experiment is probably not needed as the GOI regulator is already well known. Importantly, in reverse ChIP, DNA is synthesized; as such, there is no chromatin structure or accessibility constraints, and this will inevitably generate false binding.

### The PICh approach

PICh aims to resolve the technical problems of r-ChIP. In PICh, affinity purification relies on the specific hybridization of nucleic acid probes to selected targets and thus can be directly used to purify endogenous targets. Critically, PICh is not based on the generation of a transgenic target or the expression of adaptor proteins and does not require prior knowledge about the identity of the bound proteins (Ide and Dejardin 2015). This ensures that chromatin accessibility and structure are accounted for. Chromatin with the GOI-protein binding intact is cross-linked and broken down into smaller fragments. Next, the locus of interest is targeted with desthiobiotinylated oligonucleotide probes containing 50% of locked nucleic acids (LNAs) to improve the stability of probe–chromatin interactions. Modified nucleotides are used owing to their high melting temperature, as well as stable and specific hybridization. Hybridized probes are subsequently pulled down with streptavidin-coated beads. Because the probes are tagged with desthiobiotin, a biotin analog that binds less strongly to streptavidin, the probe–hybrid complex is easily released into solution by biotin elution, and nonspecific chromatin fragments remain bound to the beads, significantly reducing background. The final product can be analyzed by western blotting for candidate TF or MS for all GOI bound proteins (Khmelinskii and Knop 2014). PICh can be combined with stable isotope labeling by amino acids in cell culture (SILAC) for quantitative analyses (Kan et al. 2017).

Although PICh can identify proteins interacting with specific chromatin regions, there are notable limitations to consider, including the need for large amounts of cell material to obtain sufficient quantities of purified DNA-bound protein for MS, difficulties in avoiding hybridization with other genomic regions, and challenges related to the stability of TF–DNA binding, as well as reliance on repeat regions. PICh provides an enrichment factor of up to 10,000-fold; however, it is not well suited for studying the composition of single-copy small loci (∼3 kb) in mammalian genomes. Recent improvements include development of end-targeting PICh (ePICh), which allows for targeting of less abundant genomic loci by using a restriction digest to mark chromatin segment ends (Ide and Dejardin 2015). However, further development is needed for PICh to be compatible with the identification of proteins bound to a promoter of a GOI. In the future, PICh could be improved by a dCas9-based approach similar to recent work in plants (Wang et al. 2023). Enhancing cross-linking methods and developing multiplexing capabilities could also increase its efficiency and broaden its applicability for large-scale and high-resolution studies.

### Artificial chromosomes

Conventional gene expression assays often lack the native chromatin environment required to faithfully study transcriptional regulation. Plasmid-based reporters, in vitro DNA probes, or transient overexpression systems frequently fail to recapitulate higher-order chromatin structure, limiting their physiological relevance. Artificial chromosomes overcome this limitation by mimicking the behavior of endogenous chromosomes. These constructs contain the essential elements of chromosomal maintenance—replication origins, centromeres, and telomeres—enabling stable replication and segregation in host cells (Nierman and Feldblyum 2001). This architecture allows the insertion of large genomic regions, including full-length genes with their endogenous promoters, enhancers, and boundary elements, offering a substantial experimental advantage over minimal vector systems. Artificial chromosomes provide more cellular material than endogenous loci and maintain chromatin features absent from naked DNA or plasmid vectors, making them a powerful complement to methods such as reporter assays, ChIP-seq, or proteomic analysis of chromatin segments.

Yeast artificial chromosomes (YACs) were originally developed for yeast genome mapping (Burke et al. 1987) and later adapted for use in mammalian cells via cell fusion (Huxley and Gnirke 1991). However, their large size renders them difficult to transfect. Although transfection efficiency can be improved by codelivery with adenovirus (Chen et al. 1997), challenges remain with YAC isolation. Techniques such as pulsed-field gel extraction are time-consuming and low-throughput and yield limited quantities of intact YAC DNA (Larionov et al. 1996), whereas alkaline extraction methods offer modest improvements but remain low-yield (Devenish and Newlon 1982).

Human artificial chromosomes (HACs) have since been developed using either top-down approaches (truncation of native chromosomes) or bottom-up strategies (synthetic construction) (Ponomartsev et al. 2022). HACs display favorable experimental properties, including mitotic stability, episomal maintenance, and low copy number, which reduce integration-associated artifacts and make them suitable for long-term studies (Kazuki et al. 2013). HACs exhibit stable tissue-specific expression in mice and can transmit transgenes into the second generation, albeit at a modest efficiency (∼50%) (Ikeno and Hasegawa 2020). Although HAC transfer methods such as microcell-mediated chromosome transfer, whole-cell fusion, or metaphase chromosome transfer require careful optimization (Ponomartsev et al. 2022), once introduced, HACs remain extrachromosomal and avoid the risks of insertional mutagenesis or immune activation (Thomas et al. 2003).

The ability to incorporate large DNA fragments, including entire gene loci and their extended regulatory landscapes, makes artificial chromosomes uniquely valuable for interrogating gene regulation in a near-native context. They allow integration of gene reporter assays to validate regulatory sequences (e.g., promoters or enhancers) with improved control, particularly when combined with CRISPR-Cas9-mediated mutagenesis of specific elements (Nakamura et al. 2021). This has been used to study distant regulatory elements of the *CFTR* gene (Mogayzel and Ashlock 2000). In addition, the platform is amenable to standard molecular techniques: ChIP-seq can be used to map TF binding sites and chromatin accessibility within the artificial construct (Nakato and Sakata 2021), whereas PICh enables identification of chromatin-associated protein complexes bound to specific loci (Kan et al. 2017).

Artificial chromosomes also support three-dimensional chromatin conformation studies using techniques such as 3C, allowing researchers to evaluate long-range DNA interactions and chromatin architecture (Akgol Oksuz et al. 2021). Moreover, they can be used to investigate epigenetic modifications—such as histone PTMs, DNA methylation, and R-loop formation—that influence transcription. These features can be experimentally manipulated using CRISPR-based epigenetic editing tools (Nakamura et al. 2021). HACs have been used to model centromeric function through the integration of satellite DNA and the study of histone mark deposition, transcription, and kinetochore formation (Molina et al. 2016), underscoring their utility in exploring the chromatin-based regulation of any GOI.

Artificial chromosomes provide a scalable and chromatinized platform for elucidating the molecular logic of transcriptional control. By supporting high-throughput, physiologically relevant interrogation of regulatory networks at a GOI, they bridge the gap between conventional vector systems and the complex regulatory landscape of the native genome.

### CRISPR-assisted proximity labeling with APEX2

CAS9-catalyzed editing enables the modification of nucleotide sequences within the genome, facilitating a better comprehension of gene function, as well as their promoters and regulatory sequences (e.g., TF binding sites). Nevertheless, the application of the CRISPR-Cas9 system in transcriptional regulation studies goes well beyond mere DNA editing. The potential of Cas9 to inhibit transcription at various genomic locations has been investigated, given its ability to bind to any complementary sequence in the genome. Mutations were introduced into the two critical catalytic domains, RuvC and HNH, to eliminate the endonuclease activity. The resulting “dead” Cas9 (dCas9) protein still tightly binds to double-stranded DNA despite lacking the catalytic function. This inactive form of Cas9 has proven useful in studies involving programmable DNA binding (Qi et al. 2021). Through the use of customized sgRNAs, dCas9 can be directed to transcription initiation sites of any loci, where it can interfere with RNA polymerase at promoters, effectively halting transcription. Additionally, dCas9 can be targeted to the coding region of loci in a way that inhibits RNA polymerase during the elongation phase of transcription (Bikard et al. 2013).

Understanding gene expression regulation requires mapping the proteins that bind to the regulatory elements of a GOI. Traditional approaches to studying protein–DNA interactions, such as reverse ChIP and PICh, have provided valuable insights into chromatin-associated protein networks. However, these methods have limitations, particularly in detecting transient or weak interactions, which are often lost during immunoprecipitation or chromatin isolation steps. Furthermore, they rely on specific capture probes or antibodies, making them less suitable for unbiased identification of chromatin-bound proteins at a given genomic locus (Déjardin and Kingston 2009; Khan et al. 2021).To address these challenges, CRISPR-assisted proximity labeling with APEX2 (CASPEX) has emerged as a powerful method for studying chromatin-bound proteins at defined genomic locations. CASPEX utilizes a catalytically inactive mutant of Cas9 (dCas9) fused to the engineered ascorbate peroxidase (APEX2), enabling the selective labeling of proteins near a targeted DNA locus. This approach is based on the CRISPR-Cas9 targeting system, in which a sequence-specific guide RNA (gRNA) directs the dCas9–APEX2 fusion protein to a specific promoter, enhancer, or silencer of the GOI, allowing precise binding to specific genomic sequences (Myers et al. 2018). Upon activation by the addition of biotin-phenol and hydrogen peroxide, APEX2 catalyzes biotinylation of proteins within a ∼20 nm radius around the target locus. The biotinylated proteins are then captured using streptavidin affinity purification and analyzed by mass spectrometry, generating an unbiased list of chromatin-bound factors at the GOI (Hwang and Espenshade 2016). This labeling process captures dynamic protein–DNA interactions. CASPEX represents a significant advancement over existing chromatin proteomic techniques in several ways. In contrast to ChIP-MS, which requires antibodies for specific target proteins, CASPEX enables unbiased proteomic profiling of chromatin-bound proteins, even if their identity is unknown. The proximity labeling strategy allows for the detection of weak and transient interactions that are often difficult to capture using traditional immunoprecipitation-based methods (Qin et al. 2021). Another advantage is that CASPEX operates in living cells, preserving the native chromatin architecture and ensuring that the detected interactions reflect physiological conditions rather than artifacts introduced by fixation, chromatin shearing, or cross-linking (van Staalduinen et al. 2023).

This methodology has broad applications in studying gene regulation, chromatin remodeling, and TF dynamics. By allowing researchers to track changes in GOI-bound proteins under various conditions (such as cell differentiation, environmental stress, or drug treatment), CASPEX provides a useful tool for uncovering novel transcriptional regulators. It can also be applied to disease research, particularly in understanding how genetic mutations affect chromatin interactions. For example, mutations in genes associated with cancer or hematopoietic diseases often lead to altered TF binding and epigenetic dysregulation, which can now be studied in a more comprehensive manner using CASPEX (Okabe and Kaneda 2021; Morgan et al. 2022).

Another application of CASPEX is drug discovery and therapeutic target identification. By identifying the protein complexes that assemble at disease-associated regulatory elements, researchers can gain insight into potential therapeutic targets for modulating gene expression (Zou et al. 2024). Furthermore, CASPEX can be used to study long-range chromatin interactions, as modified versions of the technique have been adapted to identify proteins involved in enhancer–promoter looping and higher-order chromatin organization (Qiu et al. 2019; Ummethum and Hamperl 2020).

By combining CRISPR-Cas9-based locus targeting with high-resolution proteomic analysis, CASPEX achieves an unparalleled level of specificity and sensitivity. Unlike conventional methods, it allows for live-cell, antibody-independent, and unbiased profiling of regulatory protein complexes at specific genomic loci.

## Important consideration when analyzing regulators of GOI expression by genomic modifications

CRISPR activation (CRISPRa) and CRISPRi are techniques that employ the CRISPR-Cas9 system to regulate gene expression without permanent modifications to the DNA sequence. CRISPRa is used to enhance gene expression through a programmable dCas9 protein. This protein is combined with activation domains, such as VPR, VP64, p65, or HSF1, as a fusion construct. These domains recruit TFs, leading to increased gene transcription. This approach has been widely applied for investigating gene function (Chavez et al. 2015; Konermann et al. 2015). CRISPRi functions in the opposite manner, concentrating on silencing gene expression. This method also employs dCas9, but instead of activation domains, protein is linked to repressor domains, like KRAB. When dCas9-KRAB is directed to a gene's promoter sequence or coding regions, it binds to them and physically obstructs transcriptional complexes from accessing these areas, thereby inhibiting transcription initiation. As a result, gene expression is suppressed, enabling the study of the effects of gene silencing and determination of its role in biological processes (Qi et al. 2013; Zalatan et al. 2015).

Nevertheless, epigenetic modifications pose challenges when using CRISPR-Cas9 technology. The activity of Cas9 nuclease can influence alterations in histone modifications and DNA methylation patterns at, or near, the targeted site, especially when CRISPR is employed to regulate transcription. For instance, targeting a gene promoter using CRISPRi can result in the recruitment of repressive histone marks, leading to broader changes in chromatin accessibility (Shi et al. 2025). These epigenetic changes may extend beyond the targeted gene, influencing adjacent regions and complicating data interpretation. Moreover, the chromatin context of the target location significantly impacts CRISPR-Cas9 efficiency and specificity. For example, heterochromatin regions are less accessible to Cas9, resulting in variable editing efficiencies and possible off-target effects in regions with similar sequence compositions (Schep et al. 2024). These unintended modifications to the chromatin structure and regulatory elements can obscure the direct impact of the intended genomic modification. To mitigate these issues, researchers are developing strategies to minimize unintended epigenetic alterations. These include using high-fidelity Cas9 variants to reduce off-target effects and employing epigenome editing tools that can modify specific histone marks or DNA methylation without introducing double-strand breaks (Pei et al. 2020). Additionally, comprehensive epigenomic profiling before and after CRISPR-Cas9 experiments could help identify and account for any unintended changes in the chromatin structure.

## Summary

Understanding what regulates a GOI requires a multilayered experimental approach that integrates observational, functional, and molecular strategies. Initial RNA- and protein-centric assays provide spatial and quantitative data on transcript abundance and chromatin context. Although these techniques are informative, they remain largely correlative and require complementary validation to determine causality.

Functional assays such as CRISPR-Cas9 knock-ins offer powerful tools to create stable, endogenously tagged cell lines that faithfully report on GOI expression under native regulatory control. These systems allow dynamic monitoring of transcriptional responses and can be integrated with pooled CRISPR or siRNA libraries for high-throughput screening of candidate regulators.

To move beyond inference and identify proteins directly interacting with regulatory elements, techniques like CASPEX enable unbiased, live-cell proteomic profiling at specific genomic loci. By fusing dCas9 to APEX2, CASPEX facilitates high-resolution mapping of chromatin-associated proteins without the need for antibodies or cross-linking, capturing transient and dynamic interactions in physiological conditions.

**Figure 4.**
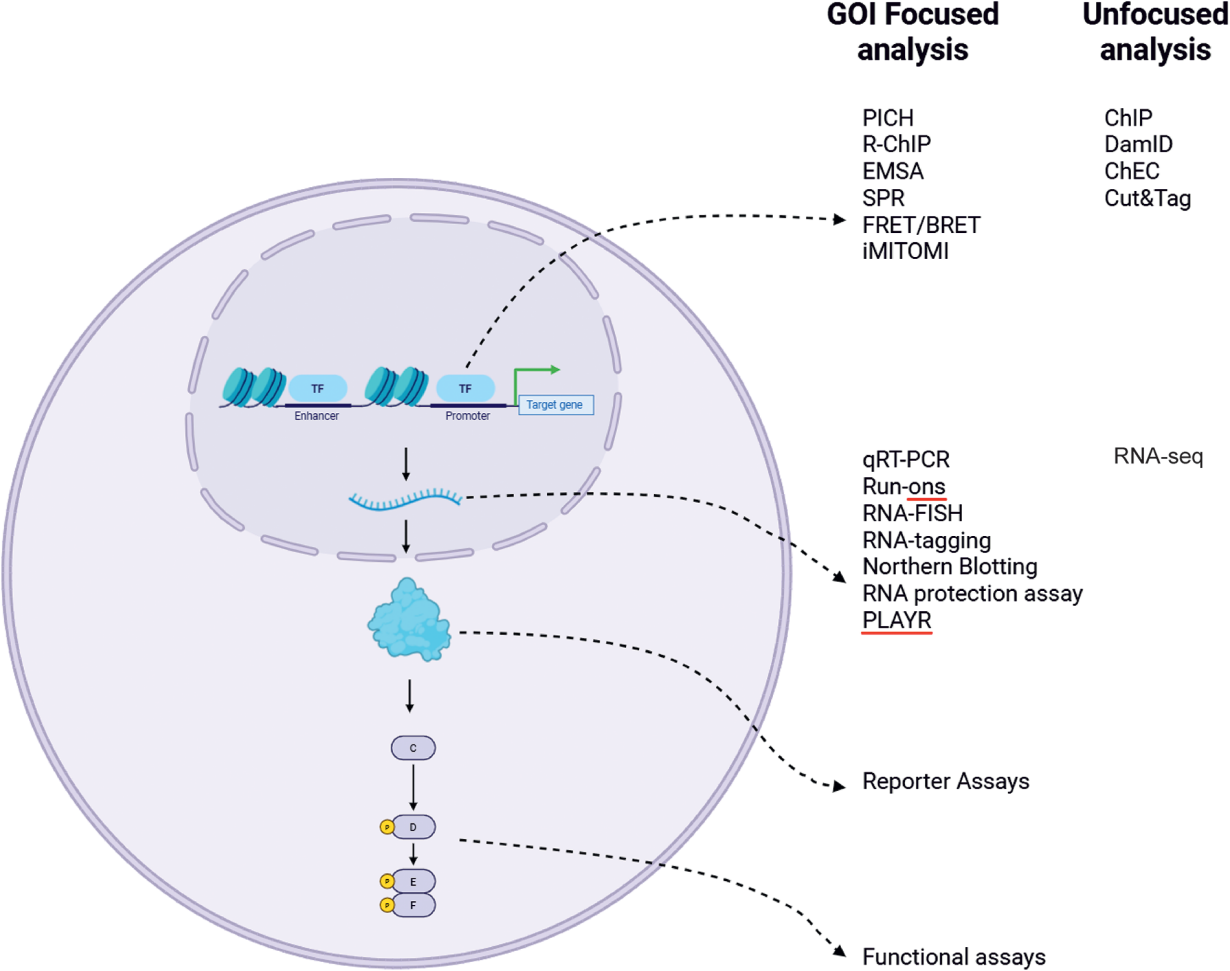
Molecular assays used to investigate gene regulatory mechanisms, broadly categorized into four functional levels: (1) transcription factor (TF)–DNA binding, (2) mRNA abundance of the GOI, (3) GOI protein expression, and (4) GOI-associated signaling networks. (1) TF–DNA binding: Assays at this level, such as ChIP-seq, CUT&RUN, or EMSAs, provide direct evidence of a physical interaction between a TF and the regulatory elements of the GOI. These approaches assess binding specificity and occupancy but do not inform on the functional consequences of such binding. (2) GOI mRNA levels: Quantification of GOI transcripts (e.g., via RT-qPCR or RNA-seq) offers a measure of transcriptional output. Changes in mRNA abundance in response to TF perturbation can suggest regulatory influence but may still reflect indirect effects mediated by intermediate factors. (3) GOI protein levels: Techniques such as reporter assay or western blotting/mass spectrometry–based proteomics assess the steady-state levels of the GOI protein. Although changes at this level may reflect transcriptional regulation, they are also influenced by post-transcriptional and translational mechanisms, including mRNA stability, translation efficiency, and protein degradation rates. (4) GOI signaling networks: This includes analysis of downstream signaling pathways or phenotypic outputs modulated by the GOI protein. These readouts are inherently multilayered and integrate regulatory events occurring at several preceding molecular levels. It is critical to recognize that as we move downstream in the gene expression cascade, from TF binding to phenotypic outcome, additional layers of regulation become increasingly prominent. For instance, using GOI protein levels as a surrogate for transcriptional regulation by TF X can be misleading. An observed increase in GOI protein levels following TF X overexpression does not necessarily imply direct TF–GOI transcriptional activation. Instead, TF X may indirectly enhance GOI protein abundance by modulating expression of genes that encode mRNA-stabilizing factors, translation enhancers, or protein chaperones that extend GOI half-life. Thus, careful interpretation of molecular readouts is essential, and conclusions regarding direct regulatory relationships should be supported by complementary mechanistic evidence across multiple levels of analysis.

**Figure 5.**
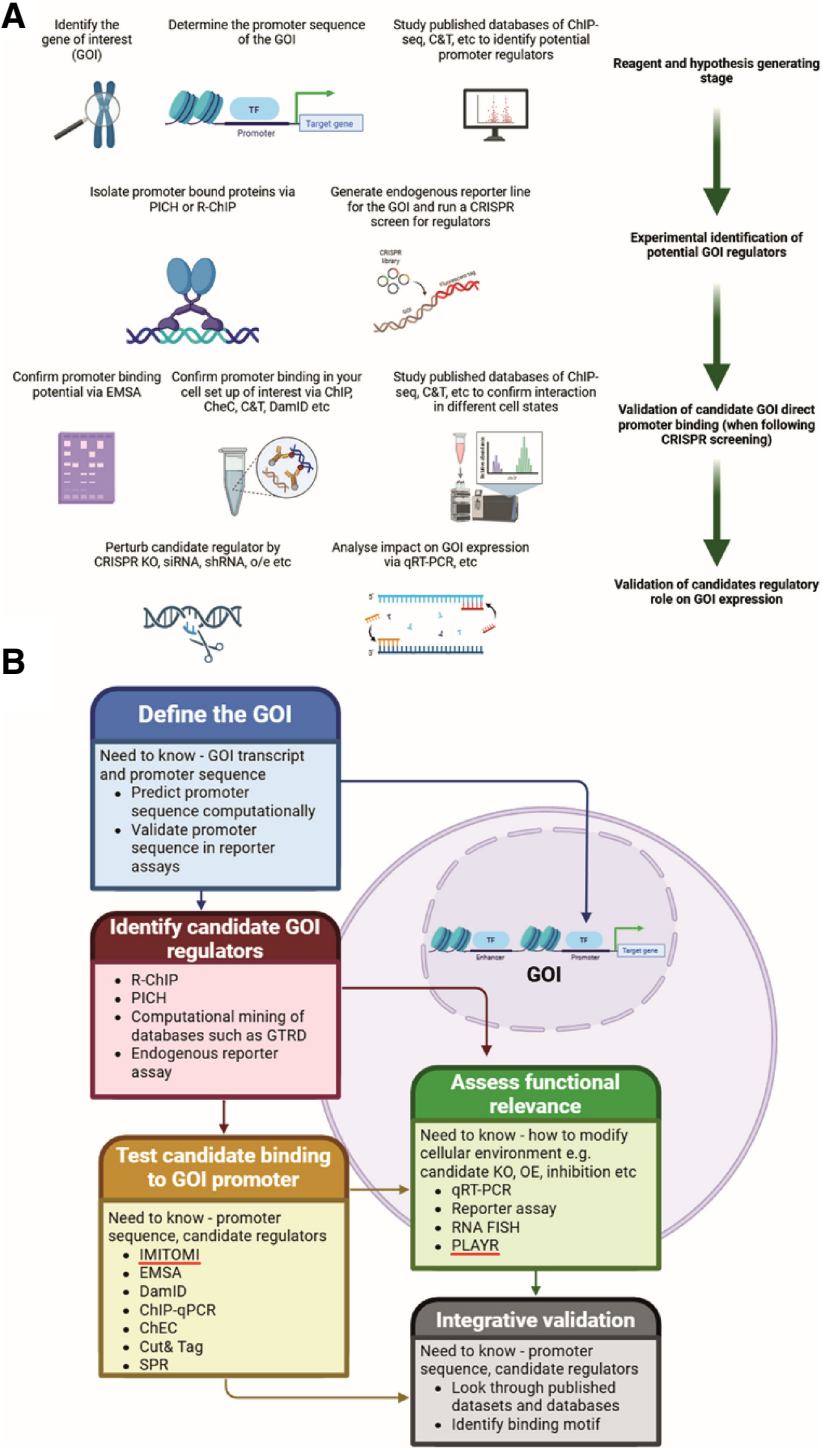
Integrated workflow for identifying direct promoter-bound regulators of a GOI. (*A*) This schematic outlines a stepwise strategy for decoding transcriptional regulation of a GOI, with a focus on identifying direct regulators acting at the promoter level. The process begins by selecting the GOI, including annotation of transcript variants, promoter structure, and determining regulatory elements. At this stage, mining publicly available data sets, such as ChIP-seq, CUT&Tag, and DamID profiles, can yield initial hypotheses regarding candidate transcription factors or chromatin regulators. Next, unbiased identification of promoter-associated proteins can be performed using locus-specific proteomics techniques such as PiCh or reverse ChIP (r-ChIP). In parallel, genome-wide CRISPR knockout screens coupled to endogenous reporters (e.g., fluorescent tagging or PLAYR-based detection) can be employed to identify both direct and indirect regulators of GOI expression. Following candidate identification, direct promoter occupancy should be validated using complementary in vitro and in vivo binding assays, including electrophoretic mobility shift assay (EMSA), ChIP, cleavage under targets and release using nuclease (CUT&RUN or CHEC), and DNA adenine methyltransferase identification (DamID), complemented by systematic evaluation of available public data sets. This step ensures that the regulators physically interact with the GOI promoter and are not acting solely via indirect pathways; consequently, it is critical to perform this analysis if candidate identification occurred via CRISPR screen as this will also identify non-promoter-bound indirect regulators. Finally, functional validation needs to be performed by perturbing candidate regulator levels—via overexpression, RNAi, or CRISPR-based approaches—and quantifying changes in GOI expression using qRT-PCR, RNA-FISH, or related transcript-level assays. Importantly, all assays should be conducted in physiologically relevant cellular contexts to preserve native chromatin architecture and regulatory interactions. (*B*) This iterative framework integrates discovery, validation, and functional characterization of promoter-bound regulatory factors and can be adapted to dissect transcriptional control of diverse genomic loci.

Together, these methods form a cohesive experimental pipeline (Fig. 4; Table 2). Observational tools prioritize candidate regulators; functional assays validate their regulatory roles; and chromatin proteomics directly identify protein–DNA interactions at the GOI locus. The integration of these strategies (Fig. 5A,B) allows for a robust, system-level understanding of transcriptional regulation in both health and disease contexts.

**Table 2.**
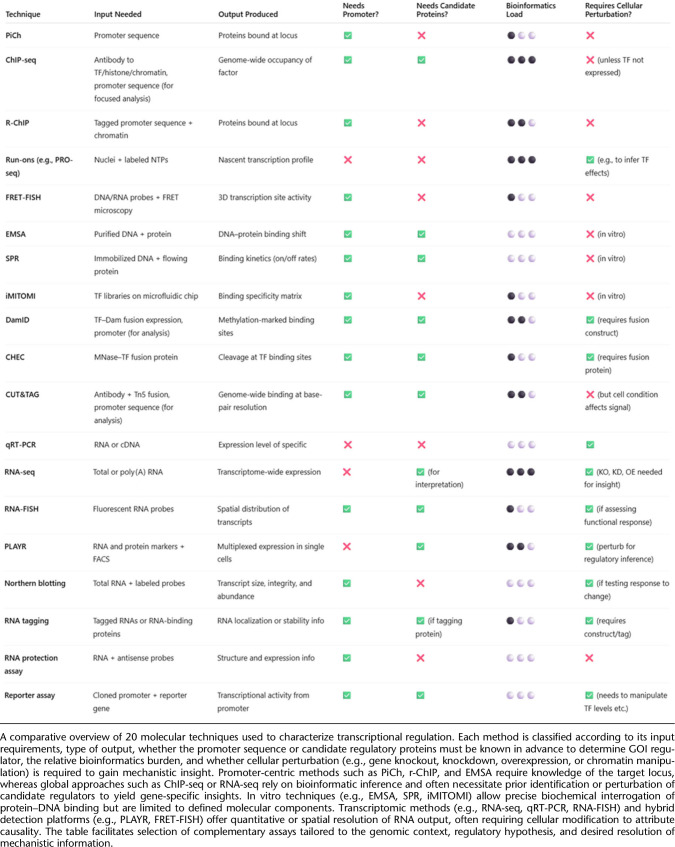
Summary of experimental approaches for identifying transcriptional regulators of a gene of interest in human cells
